# Glucose transporters in the small intestine in health and disease

**DOI:** 10.1007/s00424-020-02439-5

**Published:** 2020-08-23

**Authors:** Hermann Koepsell

**Affiliations:** Institute for Anatomy and Cell Biology, University of Würzburg, Koellikerstr 6, 97070 Würzburg, Germany

**Keywords:** Glucose transporter, Small intestine, Regulation, SGLT1, GLUT2, GLUT5, Glucose-galactose malabsorption, Fructose intolerance, Diabetes, Bariatric surgery

## Abstract

Absorption of monosaccharides is mainly mediated by Na^+^-d-glucose cotransporter SGLT1 and the facititative transporters GLUT2 and GLUT5. SGLT1 and GLUT2 are relevant for absorption of d-glucose and d-galactose while GLUT5 is relevant for d-fructose absorption. SGLT1 and GLUT5 are constantly localized in the brush border membrane (BBM) of enterocytes, whereas GLUT2 is localized in the basolateral membrane (BLM) or the BBM plus BLM at low and high luminal d-glucose concentrations, respectively. At high luminal d-glucose, the abundance SGLT1 in the BBM is increased. Hence, d-glucose absorption at low luminal glucose is mediated via SGLT1 in the BBM and GLUT2 in the BLM whereas high-capacity d-glucose absorption at high luminal glucose is mediated by SGLT1 plus GLUT2 in the BBM and GLUT2 in the BLM. The review describes functions and regulations of SGLT1, GLUT2, and GLUT5 in the small intestine including diurnal variations and carbohydrate-dependent regulations. Also, the roles of SGLT1 and GLUT2 for secretion of enterohormones are discussed. Furthermore, diseases are described that are caused by malfunctions of small intestinal monosaccharide transporters, such as glucose-galactose malabsorption, Fanconi syndrome, and fructose intolerance. Moreover, it is reported how diabetes, small intestinal inflammation, parental nutrition, bariatric surgery, and metformin treatment affect expression of monosaccharide transporters in the small intestine. Finally, food components that decrease d-glucose absorption and drugs in development that inhibit or downregulate SGLT1 in the small intestine are compiled. Models for regulations and combined functions of glucose transporters, and for interplay between d-fructose transport and metabolism, are discussed.

## Introduction

Absorption of monosaccharides in the small intestine is pivotal for caloric intake of mammalians and adjusted in accordance with food supply, food composition, and energy demand in diverse physiological and pathophysiological situations. In respect to caloric intake, d-glucose, d-galactose, and d-fructose are the most relevant monosaccharides. For absorption, monosaccharides must cross a layer of epithelial cells that are connected by tight junctions which do not allow permeation of monosaccharides [189, 192]. Because monosaccharides are hydrophilic, they cannot permeate cell membranes passively. Hence, for absorption of d-glucose, d-galactose, and d-fructose, transporters in the luminal brush border membrane (BBM) and basolateral membrane (BLM) of small intestinal epithelial cells (IECs) are required. In addition, the carbohydrate metabolism in small IECs has been adjusted to allow an adequate transcellular movement of nonphosphorylated monosaccharides.

**Fig. 1:**
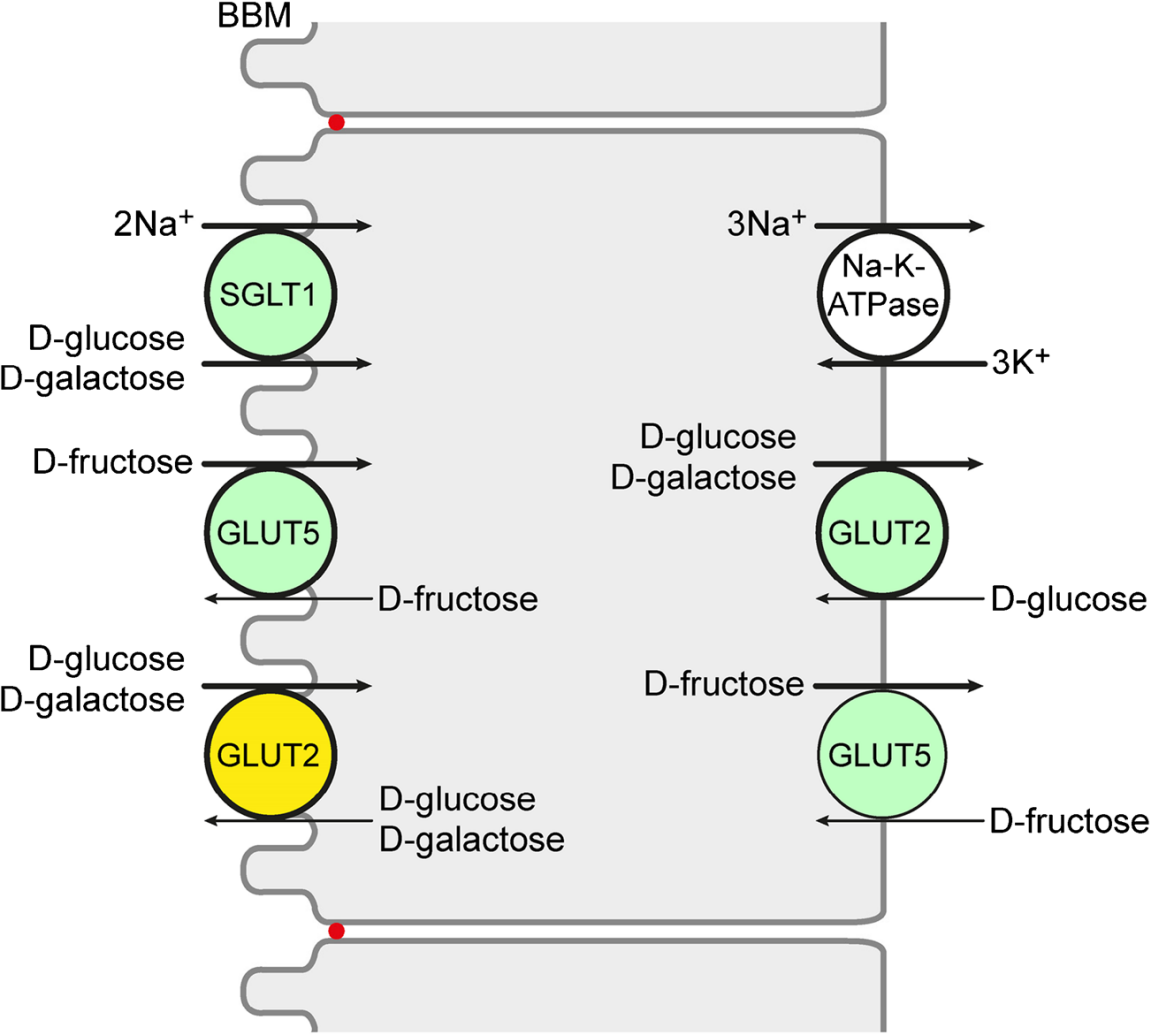
Location of monosaccharide transporters in enterocytes that are involved in small intestinal absorption of d-glucose, d-galactose, and d-fructose. The locations were determined in different species including humans. Highly expressed transporters are outlined bold. Locations of monosaccharide transporters observed under various physiological and pathophysiological conditions are indicated in green. GLUT2 that was only observed in the BBM at high small intestinal d-glucose concentrations or in some pathological conditions is indicated in yellow. The Na^+^+K^+^-ATPase in the BLM generating the inwardly directed Na^+^ gradient is also depicted

In this review, the functions and membrane locations of transporters for d-glucose, d-galactose, and/or d-fructose expressed in the small intestine are described. They belong to the *SLC2* family with facilitative diffusion transporters (GLUTs) and the *SLC5* family with Na^+^-d-glucose cotransporters (SGLTs). d-Glucose and d-galactose are transported across the brush border membrane of small intestinal enterocytes via the Na^+^-d-glucose cotransporter SGLT1 and leave the enterocytes across the basolateral membrane via GLUT2 (Fig. 1). The driving force of SGLT1-mediated monosaccharide transport is provided by the transmembrane Na^+^ gradient and membrane potential that are generated by the Na^+^-K^+^-ATPase. GLUT5 in the BBM and BLM is responsible for transport of d-fructose across the BBM and BLM (Fig. 1). At high d-glucose concentration in the small intestine, GLUT2 is also incorporated into the BBM and supports uptake of d-glucose and d-galactose across the BBM. In the next part of the review, the regulation of the most relevant small intestinal monosaccharide transporters, namely the Na^+^-d-glucose cotransporter SGLT1 and the facilitative diffusion systems for d-glucose, d-galactose, and/or d-fructose GLUT2 and GLUT5, is depicted. Therefore, the general knowledge about regulation of these transporters as well as their specific regulations in the small intestine is compiled. In addition, the combined action of the transporters for adaptation of monosaccharide absorption to different physiological conditions is discussed. Because monosaccharide transporters are also expressed in enteroendocrine cells and contribute to stimulation for enterohormone secretion, also the expression and physiological functions of monosaccharide transporters in enteroendocrine cells are reviewed.

Small intestinal monosaccharide transporters play important roles during emergence, progression, and treatment of various diseases. Covering these issues, diseases are reviewed that are caused by or associated with malfunctions of small intestinal glucose transporters. Also, current knowledge about effects of diabetes on glucose transporters in the small intestine and about the impact of small intestinal inflammations of different genesis on glucose transporters is compiled. In addition, therapeutic measures are discussed that are based on the function or change of function of small intestinal glucose transporters such as oral hydration therapy, parental nutrition, and bariatric surgery. Finally, antidiabetic food components, antidiabetic drugs, and lead compounds of antidiabetic therapy are discussed that inhibit or downregulate SGLT1 or GLUT2 in the small intestine.

## Transport mode, selectivity, and location of glucose transporters expressed in the small intestine

### Na^+^-d-glucose cotransporter SGLT1

In the small intestine of mammals, high expression of the Na^+^-d-glucose cotransporter SGLT1 (*SLC5A1*) was observed on the mRNA and protein level [424]. In the duodenum of human, rat, and mice, different relative levels of SGLT1/Sglt1 mRNA were determined following the order human > mouse > rat [195]. Proteomic analysis revealed that Sglt1 is the most abundantly expressed plasma membrane protein in mouse small intestine [422]. In mouse and human, minor expression of SGLT1/Sglt1 mRNA expression was also observed in the colon [65, 253, 431].

SGLT1/Sglt1 is a secondary active transporter that translocates one d-glucose molecule together with two sodium ions into cells employing the inwardly directed sodium gradient that is generated by the (Na^+^/K^+^)-ATPase as driving force [231]. Human SGLT1 transports d-glucose and d-galactose with respective apparent *K*_m_ values of 0.5 mM and 1 mM at physiological membrane potential and inward-directed Na^+^ gradient, whereas it does not interact with d-fructose (Table 1) [424]. SGLT1/Sglt1-mediated uptake is inhibited by phlorizin but not by phloretin and cytochalasin B [424].

**Table 1:** Characteristics of uptake of d-glucose, d-galactose, and/or d-fructose by human SGLT1, GLUT2, GLUT 5, and GLUT7

Transporter	Approximate *K*_m_ value (mM)			Reference
	d-Glucose	d-Galactose	d-Fructose	
SGLT1	0.5	1	n.i.	[424]
GLUT2	17	92	76	[182]
GLUT5	n.d.	n.i.	6	[46]
GLUT7	0.3	n.i.	0.06	[236]

*n.i.*, no interaction; *n.d.*, not determined

SGLT1*/*Sglt1-related immunoreactivity was detected in BBMs of IECs and in enteroendocrine cells [25, 132, 142, 155, 253, 387, 410]. Apart from some differences in segment distribution, similar membrane location of SGLT1/Sglt1 was observed in different species. In human IECs, SGLT1 protein was observed not only in the BBM but also in subapical vesicles [410]. Similarly, in differentiated CaCo-2 cells, a model of human enterocytes, SGLT1, was localized to the BBM and intracellular vesicles [198]. The intracellular location of SGLT1/Sglt1 in enterocytes is consistent with data revealing a d-glucose-dependent regulation of the exocytotic pathway of SGLT1/Sglt1 in the small intestine of mouse and humans [66, 341, 408]. In rats, the BBM abundance and transport capacity of Sglt1 per unit length in the jejunum was higher compared to that in the duodenum and ileum [25, 83, 142, 203]. These differences are mainly due to the different length of the villi and microvilli. Whereas in BBMs along the small intestinal villi very strong SGLT1/Sglt1-related immunoreactivity was observed, only weak or no staining was detected in BBMs of enterocytes in the crypts [25, 30, 83, 142, 168, 231]. This is consistent with the observation that enterocytes are dividing within the crypts and differentiate during migration along the villi [109, 112]. In human and rat, similar abundance of SGLT1/Sglt1-related immunoreactivity was observed in BBMs of jejunal enterocytes of female and male [25, 410].

In mouse and human small intestine, SGLT1/Sglt1-related immunoreactivity was also detected in enteroendocrine K cells secreting glucose-dependent insulinotropic hormone (GIP) and in L cells secreting glucose-dependent secretion of glucagon-like peptide 1 (GLP-1) [132, 410].

### Glucose facilitator GLUT2

In the small intestine of mammals and rodents, also high expression of GLUT2/Glut2 (*SLC2A2*) was observed in most species [123, 394, 431]. The abundance of GLUT2/Glut2 mRNA in the duodenum of human, mouse, and rat follows the order rat > mouse >> human [195]. In mice, a similar abundance of Glut2 mRNA was observed in the jejunum, ileum, and colon [345].

GLUT2/Glut2 is a facilitated diffusion system that transports d-glucose, d-galactose, and fructose in human with apparent *K*_m_ values of ~ 17 mM, ~ 92 mM, and ~ 76 mM, respectively (Table 1) [133, 182]. GLUT2/Glut2-mediated transport is inhibited by phloretin and cytochalasin B but not by phlorizin. Of note, human GLUT2 transports d-glucosamine with an apparent *K*_m_ of ~ 0.8 mM [403].

Between meals when the glucose concentration in the small intestinal lumen is low or in human mucosa biopsies that were taken after food traces had been removed by bowl rinsing, GLUT2/Glut2-related immunoreactivity was observed at the BLM of small intestinal enterocytes [70, 98, 345, 395]. In membrane vesicles derived from BLMs of rat small intestine, glucose transport with properties similar to Glut2 was observed [63]. In the presence of high glucose concentrations, in various species, GLUT2/Glut2-related immunoreactivity was also detected in the BBM and phloretin- or gluosamine-inhibited glucose uptake was detected in BBM vesicles [5, 73, 132, 134, 189–191]. In addition, phloretin- or gluosamine-inhibited glucose uptake was detected in BBM vesicles [132, 189].

### Fructose facilitator GLUT5

In the small intestine of mammals and rodents, also GLUT5/Glut5 (*SLC2A5*) is expressed abundantly [46, 92, 188, 195]. Similar for SGLT1/Sglt1 and GLUT2/Glut2, species differences were observed for the abundance of GLUT5/Glut5 mRNA in the duodenum following the order rat > human >> mouse. In human, mRNA abundance of GLUT5 is higher in the jejunum compared to that in the ileum [46].

In human and rodents, GLUT5/Glut5 is an efficient facilitative diffusion system which is specific for d-fructose [46, 266, 311]. Fructose uptake by GLUT5/Glut5 is not inhibited by phlorizin, phloretin, and cytochalasin B. Human GLUT5 transports d-fructose with an apparent *K*_m_ of 6 mM (Table 1) [46].

In human and rodents, GLUT5/Glut5 was localized to the BBM of small intestinal enterocytes [28, 46, 70, 81, 254, 358]. Of note, in one study, GLUT5-related immunoreactivity was also detected in the basolateral membrane of human enterocytes [36].

### Glucose facilitators with nonresolved functional significance

#### GLUT1

In the small intestine of human, mice, and rat, mRNA of the erythroid glucose facilitator GLUT1/Glut1 (*SLC2A1*) was detected [59, 122, 195, 334, 402, 431]. In rat with streptozotocin (STZ)-induced diabetes, Glut1-related immunoreactivity was observed in the BLM and the BBM of small intestinal enterocytes [39]. After duodeno-jejunal bypass (DJB) in rats, Glut1 in the BLM and basolateral uptake of 2-deoxy-2[^18^F]fluoro-d-glucose (2-[^18^F]DG) in the alimentary jejunal limb were higher compared to the respective jejunal segment of sham-operated animals [59, 334]. Because Glut1 was not detected in the small intestine of nondiabetic rats [39], it is not supposed to contribute significantly to d-glucose absorption in healthy individuals.

#### GLUT7

In human small intestine and colon, mRNA of GLUT7 (*SLC2A7*) was detected [236]. Human GLUT7 transports D-glucose and D-fructose with apparent *K*_m_-values of 0.3 mM and 0.06 mM, respectively, but does not accept D-galactose as substrate (Table 1) [236]. Because Glut7 related immunoreactivity was located to the BBM of rat small intestinal enterocytes [236] it could be relevant for fructose absorption at low fructose concentrations.

#### GLUT8

In the small intestine and colon of mice and in CaCo-2 cells, expression of Glut8 (*SLC2A8*) was observed [89, 169, 326, 327, 381]. In IECs and after expression of GLUT8/Glut8 in different cell types, GLUT8/Glut8 was located to intracellular vesicles [169, 243, 327]. Different to the small intestine, plasma membrane location of Glut8 in blastocytes was promoted by insulin [19, 169, 243, 307, 344]. After expressing a GLUT8 mutant with an inactivated N-terminal dileucine motif in oocytes, uptake of 2-deoxy-d-glucose (2-DOG) with an apparent *K*_m_ of 2.3 mM was measured [169]. Different to wild-type mice, the abundance of Glut12 in enterocytes of Glut8-knockout mice was increased in response to a high-fructose diet [84]. Based on these observations, the hypothesis was raised that GLUT8/Glut8 interacts with GLUT12/Glut12. The functional role of GLUT8/Glut8 in the small intestine is enigmatic.

#### GLUT12

In human small intestine, GLUT12 (*SLC12*)-related immunoreactivity was observed in a Western blot [323]. After expression in *Xenopus* oocytes, GLUT12-mediated uptake of 2-DOG was demonstrated that was inhibited by d-fructose and d-galactose [324]. In mice in which Glut2 was overexpressed, the absorption of d-fructose in the small intestine was increased 2.5-fold [84]. After expression of GLUT12 in Chinese hamster ovary cells, the transporter was localized to the Golgi and the plasma membrane [117]. In human skeletal muscle cells, a N-terminal dileucine motif corresponding to the abovementioned dileucine motif in GLUT8 was required for insulin-dependent changes of GLUT12 abundance in the plasma membrane [4, 117, 377]. Further studies are required to elucidate the functional role of GLUT12/Glut12 in the small intestine.

#### SGLT4

SGLT4 (*SLC5A9*) has been cloned from human [391]. Expressing human SGLT4 in COS-7 cells the authors observed Na^+^-dependent AMG uptake with an apparent *K*_m_ value of 2.6 mM that could be inhibited by high concentrations of d-glucose, d-fructose, and d-galactose. It was observed that human SGLT4 is highly expressed in the small intestine and to lower degrees in pancreas, skeletal muscle, lung, kidney, caecum, colon, and testis [65]. Expression of Sglt4 was also observed in mouse small intestine [31]. So far, plasma membrane location of SGLT4/Sglt4 in IECs has not been determined. Also, transport of d-glucose, d-fructose, and d-galactose by SGLT4/Sglt4 has not been demonstrated and characterized. Hence, the relevance of SGLT4/Sglt4 for absorption of d-glucose, d-fructose, and d-galactose has not been resolved.

## Regulation of monosaccharide transport in the small intestine

### Nonspecific and specific regulations

In the small intestine, nonspecific and specific adaptations are developed to cover different energetic demands for monosaccharide absorption [112]. In nonspecific adaptations, small intestinal capacity for uptake of different monosaccharides and nutrients is changed in parallel, whereas specific adaptations affect uptake of individual or few monosaccharides. In nonspecific adaptations, the overall absorptive capacity of the small intestine is changed. This includes changes of the absorptive surface, the number of the enterocytes (hyperplasia), their size (hypertrophy), and the degree of their differentiation. The absorptive surface is determined by small intestinal length, height of villi, and length of microvilli. Enterocyte stem cells located within the crypts divide and migrate onto the villi. They differentiate during migration and are exfoliated at the top of the villi exhibiting life spans between days and weeks [112]. Nonspecific adaptations of the small intestine are slow and require few days at minimum. They have been observed in response to changed nutrition [361], during diabetes [246, 343], and after surgical interventions [40, 136].

Specific adaptations of monosaccharide absorption include changes in the amount of transporter molecules in the BBM and/or BLM of IECs. They may be due to transcriptional and/or posttranscriptional regulations of individual transporters and may occur within minutes, hours, or days. Specific regulations of monosaccharide transporters have been observed following a diurnal rhythm, directly after uptake of carbohydrate-rich meals, in response to carbohydrate content of diets, in response to hormones and neuronal activation, accompanied with diseases, and after surgical interventions. Since regulatory signals such as carbohydrates and hormones may regulate different glucose transporters, coordinated regulations of transporters in the BBM and BLM are possible. Current knowledge about regulations of small intestinal transporters for d-glucose, d-galactose, and d-fructose in humans is fragmentary for several reasons. Thus, only some of the potentially involved regulatory mechanisms have been investigated and only very few investigations have been performed with human small intestine. All in vivo measurements were carried out in rodents. Additional investigations were performed in cultivated cells derived from porcine kidney (LLC-PK1 cells) or from human intestinal tumors (Caco2 cells) or after expression of transporters in cultivated epithelial cells or oocytes. In Table 2, a survey about the investigated regulatory mechanisms, species, and employed methods are presented.

**Table 2:** Description of the studies reported in this review in which regulations of monosaccharide transporters were investigated that are supposed to be relevant for absorption of d-glucose, d-galactose, and d-fructose in human small intestine

Level of regulation	GLUT2/Glut2	GLUT5/Glut5	SGLT1/Sglt1
Transcription	Human: e.c.c.; rodents: in vivo	Human: e.c.c., C; rodents: in vivo	Human: e.c.c., C; pig: L; sheep: in vivo; rabbit: e.c.c.; rodents: in vivo
mRNA stability		Human: C	Pig: L
Translation or protein stability	Rodents: in vivo (n.d.)	Rodents: in vivo (n.d.)	Sheep: in vivo (n.d.); rabbit: e.o. (p.s.)
Protein trafficking	Rodents: in vivo		Human: e.o.; rabbit: e.o.; rodents: in vivo

Different levels of regulation were investigated in different species in vivo and in vitro. For in vitro studies, two cell models and different expression systems were employed

*C*, measurements in Caco2 cells derived from human; *L*, measurements in LLCP-K1 cells derived from pig; *e.o.*, transporter expression in oocytes of *Xenopus laevis*; *e.c.c.*, transporter expression in cultivated cells; *n.d.*, no differentiation between translation and protein stability was performed; *p.s.*, protein stability was investigated

### Regulation of SGLT1

#### Basic knowledge about transcriptional regulation in human

**Fig. 2:**
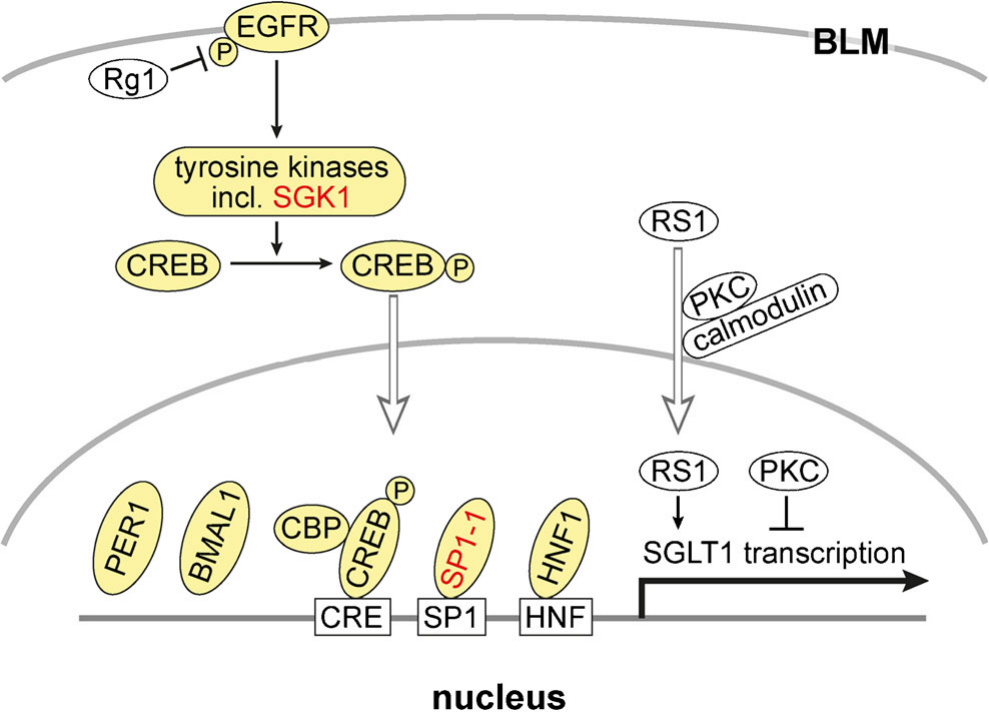
Transcriptional regulation of SGLT1/Sglt1. Response elements in the promotor of human SGLT1 and components that were shown to be involved in transcriptional regulation of SGLT1/Sglt1 are indicated. Components that participate in transcriptional regulation in the small intestine are marked with yellow. If they participate in d-glucose-dependent regulation, they are indicated in red. EGFR, epithelial growth factor receptor; Rg1, ginsenoside component Rg1; SGK, serum- and corticoid-stimulated kinase; CREB, cAMP response element- binding protein- ; CBP, cAMP response element protein–binding protein; PER1, period circadian regulator 1; BMAL1, brain and muscle ANRT-like 1; SP1-1, specificity protein 1 subtype 1; HNF1, hepatic nuclear factor 1

Several factors that regulate the transcription of human SGLT1 at the promotor region have been identified (Fig. 2). For example, binding of hepatic nuclear factor (HNF) 1, transcription factor SP1, and cAMP response element–binding protein (CREB) to the 5′ region of the human SGLT1 gene and their effects on transcription have been demonstrated [259, 417]. Employing murine STC-1 cells derived from intestinal endocrine cells, data were obtained which suggest that glucose-induced upregulation of transcription of ovine SGLT1 is dependent on the integrity of the HNF-1 consensus sequence in the promotor [404]. Binding of transcription factor HNF-1β to the promotor of rat SGLT1 has been demonstrated [319]. In Chinese hamster ovary cells transfected with rabbit Sglt1, transcription was stimulated after inhibition of protein kinase C [57].

### Basic knowledge about transcriptional and posttranscriptional regulation derived from studies with LLC-PK1 cells

In LLC-PK1 cells that are derived from porcine kidney, expression and function of endogeneous SGLT1 is upregulated during confluence [12, 267]. This may be considered a model for upregulation of SGLT1 in the small intestine during differentiation of enterocytes. In subconfluent LLC-PK1 cells, SGLT1 mRNA abundance, glucose uptake, and PKC activity are low whereas in confluent LLC-PK1 cells, SGLT1 mRNA abundance, glucose uptake, and PKC activity are high [12, 205, 267, 353]. When PKC is blocked in confluent cells, dedifferentiation is induced and expression SGLT1 mRNA and SGLT1 protein is decreased. Dedifferentiation and decreased SGLT1 expression is also induced when confluent LLC-PK1 cells are incubated with polyamines, whereas differentiation combined with an increase of SGLT1 abundance is promoted when synthesis of putrescine is blocked by inhibition of ornithine decarboxylase (ODC) [302]. Regulatory protein RS1 (*RSC1A1*) [222, 406] is involved in confluence-dependent regulation of SGLT1 transcription [205] (Fig. 2). RS1 has been shown to affect expression of human SGLT1 in bacteria (R. Poppe and H. Koepsell, unpublished data). Porcine SGLT1 exhibited a 5-fold higher nuclear abundance in subconfluent compared to confluent LLC-PK1 cells [115, 205]. Nuclear abundance of RS1 is regulated via a nuclear shuttling domain that contains a Ca^2+^-dependent calmodulin binding site and is associated with a PKC-dependent phosphorylation site, and nuclear abundance of RS1 is modulated by PKC [115] (Fig. 2).

The higher overall abundance of SGLT1 mRNA in confluent versus subconfluent LLC-PK1 cells associated with decreased PKC activity and increased PKA activity is partially due to effects of PKC and PKA on the stability of SGLT1 mRNA [303, 353]. In LLCP-K1 cells, two transcripts of SGLT1 mRNA with 2.2 and 3.9 kilobases (Kb) were observed which differ in length of the 3′ untranslated region. Whereas PKC decreases the stability of both transcripts [353], cAMP and PKA stimulate the stability of the 3.9-kb transcript [232, 303]. The cAMP-induced increase of mRNA stability is due to binding of human antigen R (HuR), an RNA-binding protein of the embryonic lethal abnormal vision family, to 47 nucleotides in a 120 nucleotide long uridine-rich element (URE) in the 3′ untranslated region of the 3.9-kb SGLT1 transcript [244]. Expression of HuR was increased by cAMP and binding of HuR was increased by cAMP-dependent phosphorylation of non-identified cellular proteins. Of note, cytoplasmic HuR levels are regulated by AMP-stimulated protein kinase (AMPK), which is stimulated by metformin [413, 440].

When confluent LLC-PK1 cells were cultivated with 25 mM versus 5 mM d-glucose in the medium, the transcription of SGLT1 was decreased after 2 days [205, 284]. The glucose-dependent regulation of SGLT1 in LLC-PK1 cells differs from the glucose-dependent regulation of SGLT1 in the small intestine because it goes into the opposite direction and appears to be independent of RS1 [205].

**Fig. 3:**
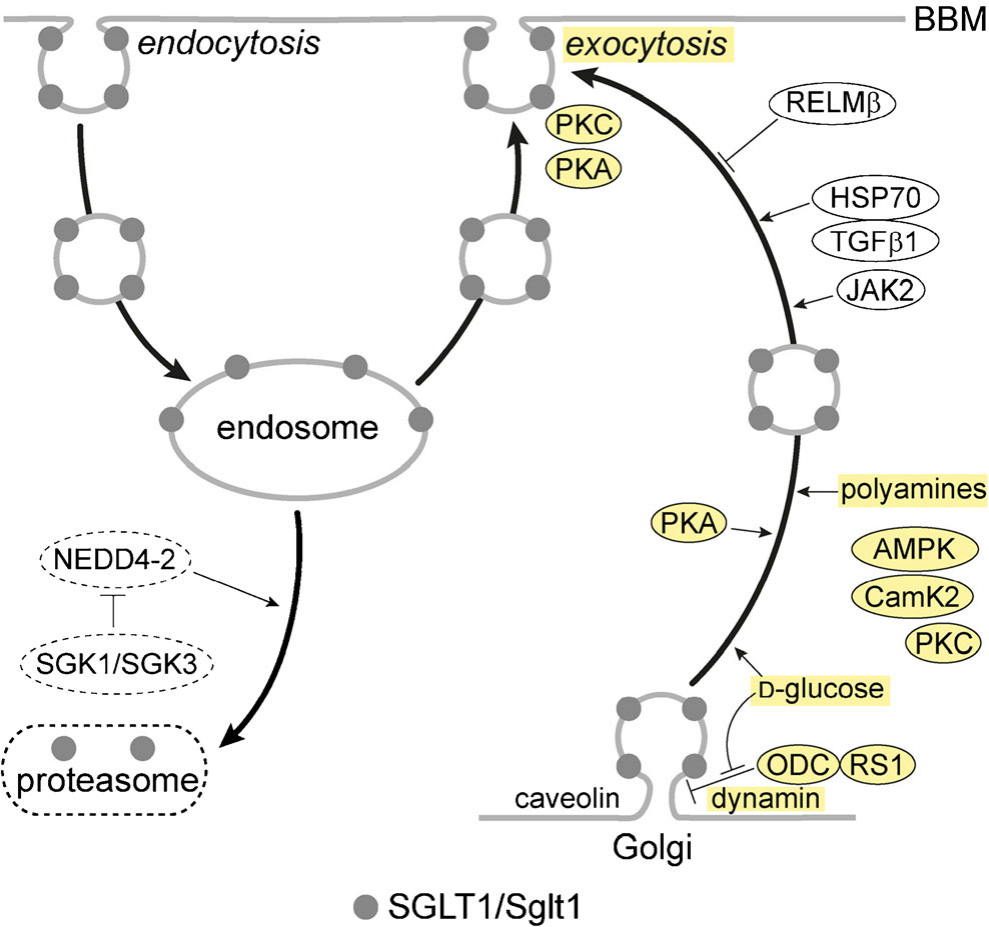
Membrane trafficking of SGLT1/Sglt1. The scheme is based on experiments in which SGLT1 from human or rabbit was expressed in oocytes, and on experiments with mouse small intestine. Components which have been shown to be involved in short-term regulation of SGLT1/Sglt1 in the small intestine are indicated in yellow. The proteasomal degradation pathway is not well explored. RELM, resistin-like molecule; HSP, heat shock protein; TGF, tissue growth factor; JAK, Janus-activated kinase; AMPK, AMP-activated protein kinase; CamK, calmodulin-stimulated kinase; ODC, ornithine decarboxylase; NEDD, neural precursor cell expressed developmentally downregulated; SGK, serum- and glucocorticoid-stimulated kinase

After a 2 h incubation of confluent LLC-PK1 cell at 42 °C, cytosolic heat shock protein (HSP) 70 and SGLT1-mediated glucose uptake were increased. The upregulation of SGLT1 activity was mediated by tissue growth factor beta 1 (TGFβ1) and associated with translocation of SGLT1 and HSP70 to the apical cell side [171, 383]. The data suggest that the exocytotic pathway of SGLT1 is influenced by TGFβ1 and may be activated by HSP70 (Fig. 3).

#### Basic knowledge about posttranscriptional regulation derived from studies with oocytes of *Xenopus laevis*

Studies in which human SGLT1 or human SGLT1 fused to yellow fluorescent protein (YFP-SGLT1) was expressed in *Xenopus laevis* oocytes were employed to characterize short-term regulations of SGLT1 abundance in the plasma membrane. Oocytes expressing SGLT1 were incubated for short time periods with membrane permeant modifiers and/or injected with various compounds, and effects on transport or plasma membrane abundance of the transporter were analyzed. The oocytes were incubated with PKA or PKC [156, 407] and/or injected with inhibitors of endocytosis or exocytosis [407], with brefeldin A that destroys the Golgi, with protein RS1 (*RSC1A1*), or with peptides derived from RS1 [341, 405, 408]. After time periods up to 60 min, SGLT1-mediated uptake of α-methyl-d-glucoside (AMG) or AMG-induced currents, membrane capacitance reflecting the plasma membrane surface area, or plasma membrane abundance of YFP-SGLT1 was measured. Upregulation of AMG-mediated inward currents and increase of plasma membrane capacitance were observed several minutes after stimulation of SGLT1 expressing oocytes with PKA or PKC [156, 407]. Because no short-term changes in SGLT1 activity were detected when endocytosis was inhibited by imipramine or chlorpromazine whereas short-term downregulation was observed after inhibition of exocytosis by botulinum toxin B or after destroying the Golgi with brefeldin A [104, 407], the exocytotic pathway is supposed to be mainly involved in short-term regulation of SGLT1 (Fig. 3). The exocytotic pathway of SGLT1 was accelerated by Janus-activated kinase (JAK) [166] and shown to be dependent on dynamin and caveolin 1 [104, 407]. Evidence was provided that protein RS1 is critically involved in regulation of the dynamin-dependent exocytotic pathway of SGLT1 by decelerating the release of SGLT1 containing vesicles from the Golgi [407] (Fig. 3). Thus, downregulation of human SGLT1 by injection of RS1 or the RS1-domain RS1-Reg was abolished when the Golgi was destroyed with brefeldin A [407, 408], and RS1 and porcine SGLT1 were colocated at the *trans*-Golgi network (TGN) in LLC-PK1 cells [212]. RS1-Reg contains multiple consensus sequences for protein kinases, and the affinity for downregulation of human SGLT1 by RS1-Reg was increased after activation of PKC or calmodulin-stimulated kinase 2 (CamK2). This suggests that kinases modulate RS1-Reg-mediated short-term regulation of SGLT1 [408]. Short-term regulation of SGLT1 abundance by RS1-Reg is d-glucose-dependent and involves the interaction of RS1 with ODC. Since d-glucose-dependent regulation has been shown to be relevant for the small intestine, it is described in the next chapter.

Proteasomal degradation of rabbit SGLT1 after ubiquitination by the ubiquitin ligase Nedd4-2 has been shown to be blunted by the serum- and glucocorticoid-inducible kinases SGK1 and SGK3 (Fig. 3) [87].

#### Transcriptional regulation in the small intestine by epithelial growth factor

Epithelial growth factor (EGF) activates transcription of SGLT1/Sglt1 in the small intestine [417] (Fig. 2). EGF binds to the EGF receptor (EGFR) that is supposed to be localized in the BLM of enterocytes [308]. Binding of EGF to EGFR stimulates phosphorylation of cAMP response element–binding protein (CREB) by tyrosine kinases including serum- and corticoid-stimulated kinase (SGK) 1 (Fig. 2). After phosphorylation, CREB migrates into the nucleus and activates SGLT1/Sglt1 transcription by binding to cAMP response element (CRE) in the promotor. This activation of transcription is modulated by cAMP response element protein–binding protein (CBP). In the small intestine of diabetic *db/db* mice, stimulation of Sglt1 expression by SGK1 was demonstrated [240].

#### Diurnal regulation in the small intestine

Rats kept with free access to food and 12-h lightening ingest and absorb ~ 90% of their daily food and have a higher capacity for glucose absorption during night time [116, 125, 374]. The capacity for glucose absorption peaks in the late light/early dark phase. During night time, also an increase of sucrase activity was observed [148]. The higher glucose uptake and sucrase activity during night time were independent from lightening schedule [280, 373] and persisted in starved animals [124, 375].

Consistent with the notion that SGLT1/Sglt1 is rate limiting for glucose absorption [132], circadian rhythmicity peaking in the late light/early dark phase was also observed in rodents for abundance and transcription of Sglt1 mRNA, for abundance of Sglt1 protein, and for Sglt1-mediated glucose transport [69, 177, 319, 389, 390]. Since in the small intestine of monkeys different SGLT1 mRNA abundance was observed at 9 a.m. versus 10 p.m. [319], also humans are supposed to exhibit a circadian periodicity of small intestinal SGLT1 expression.

Limited information about the potential molecular mechanism for rhythmic regulation of SGLT1/Sglt1 transcription is available. Transcription factors HNF-1α, HNF-1β, histone acetylation, an mRNA elongation factor, and clock genes appear to be involved. In rats fed at libitum, binding of transcription factors HNF-1α and HNF-1β to the promotor of Sglt1 was different at 10 a.m. versus 4 p.m. [319]. In mice, it was observed that the circadian expression of Sglt1 mRNA was associated with histone acetylation and mRNA abundance of elongation factor BRD4-P-TEFb [428]. Evidence was provided that the clock gene products, brain and muscle arnt-like protein (BMAL) 1), and period 1 protein (PER1), are involved in the diurnal regulation of Sglt1 transcription [23, 177] (Fig. 2).

Like small intestinal glucose absorption, circadian periodicity of Sglt1 transcription occurs independently of food intake. Since the diurnal changes of Sglt1 mRNA abundance and Sglt1-mediated glucose uptake precede food uptake, they may be considered anticipatory to food ingestion [389]. The food-independent periodicity of glucose absorption and Sglt1 expression is accompanied and probably controlled by neuroendocrine regulation involving insulin. Thus, diurnal changes of blood insulin concentration and of insulin sensitivity independently of feeding were abolished after truncation of the vagus nerve [34, 147, 248]. The diurnal food-independent periodicity of Sglt1 mRNA abundance is supposed to be mainly due to changes of Sglt1 transcription; however, regulation of mRNA degradation may be also involved. Noteworthy, the diurnal changes of Sglt1 mRNA were observed in enterocytes at the upper villi [389]. Since enterocytes need 1 to 2 days to migrate from crypts where they divide to villi, the diurnal changes of Sglt1 mRNA must occur in differentiated enterocytes on the villi.

#### Short-term post-translational regulation in the small intestine by glucose

Glucose-dependent, short-term upregulation of SGLT1/Sglt1 in the small intestinal BBM has been observed in rodents and humans. In rat, the *V*_max_ of phlorizin-inhibited glucose uptake across the small intestinal BBM was increased when the intestine had been perfused for 30 min with buffer containing 25 mM of d-glucose [351]. In mouse small intestine, *V*_max_ of phlorizin inhibited AMG uptake into BBM vesicles and Sglt1 protein in the BBM were increased 30 min after the animals had been gavaged with d-glucose [132].

**Fig. 4:**
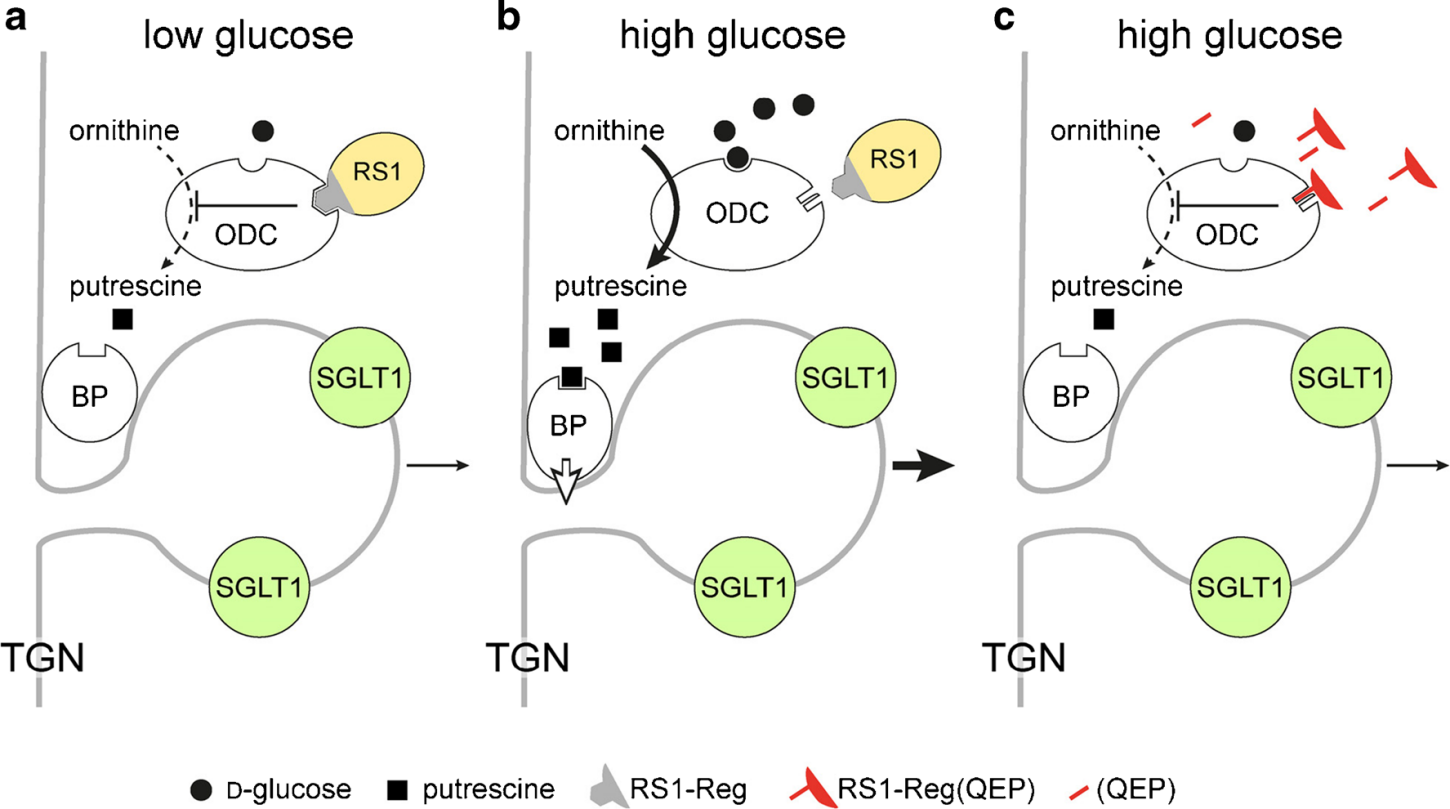
Model depicting the presumed role of RS1 in d-glucose-dependent release of vesicles containing human SGLT1 from the Golgi (a, b) and the action of modified peptides derived from RS1-Reg (c) that downregulate SGLT1 in the BBM. RS1-Reg in RS1 is indicated in gray. TGN, *trans*-Golgi network; ODC, ornithine decarboxylase; BP, putrescine-binding protein of a budding protein complex that induces the release of vesicles containing human SGLT1 from the TGN

Effects of glucose on posttranscriptional and post-translational regulation of human SGLT1 expressed in oocytes were studied in detail [65, 341, 405, 407, 408]. On the basis of these data, hypotheses on glucose-dependent short-term regulation of human SGLT1 were raised (Fig. 3). Accordingly, d-glucose-dependent upregulation of SGLT1 in the plasma membrane is due to a glucose-induced acceleration of the exocytotic pathway of SGLT1 from the Golgi that is modulated by protein RS1 (*RSC1A1*) [66, 407, 408]. Human SGLT1 or YFP-SGLT1 was expressed in oocytes, and the post-translational active domain of RS1 (RS1-Reg) or peptide motifs of RS1-Reg were injected into the oocytes. The injections were performed without or with coinjection of non-metabolizable AMG, and effects on SGLT1 in the plasma membrane were analyzed [66, 341, 405, 407, 408]. In the experiments, either AMG uptake, AMG-induced inward currents, or fluorescence of YFP-SGLT1 in the plasma membrane was determined. Since RS1-Reg contains multiple consensus sequences for phosphorylation including the functional active motif Gln-Ser-Pro (QSP) [405, 408], experiments were also performed with QSP, Gln-Glu-Pro (QEP), and with RS1-Reg mutants in which serine residues in potential phosphorylation sites were mutated. Additional investigations that were performed with mouse small intestine, human small intestinal mucosa, and differentiated Caco2 cells, a model for human small intestinal enterocytes, revealed that RS1-Reg-mediated glucose-dependent regulation of SGLT1/Sglt1 occurs in the small intestine [66, 408] (C.Otto et al. 2020, unpublished data). Recently, further details about the mechanism of the RS1-Reg-mediated regulation of human SGLT1 at the TGN were enlightened [66]. Thus, evidence was obtained that RS1-Reg binds to the ODC which has been assigned to several intracellular locations but appears to be also located at the Golgi [185]. It was detected that RS1-Reg inhibits the enzymatic activity of ODC, i.e., the formation of putrescine by decarboxylation of ornithine, in a glucose-dependent manner, and that ODC contains a glucose-binding site. In addition, evidence was presented that downregulation of human SGLT1 abundance in the plasma membrane can also be induced by inhibiting ODC activity, and that this effect is counteracted by putrescine. Based on these data, hypotheses have been raised and included in a model (Fig. 4(A, B)) [200]. At low intracellular d-glucose concentration, the RS1-Reg domain of RS1 binds to ODC and inhibits ODC activity reducing the local concentration of putrescine at the TGN. Under this condition, dynamin-dependent budding of SGLT1 containing vesicles from the TGN mediated by non-identified budding proteins that interact with putrescine is slow. At high intracellular d-glucose concentration, d-glucose binds to ODC and induces a conformational change that leads to a dissociation of bound RS1 and results in activation of ODC activity. As a result, the local concentration of putrescine at the TGN is increased and putrescine binds to the budding protein complex and activates budding activity of the complex.

#### Short-term post-translational regulation in the small intestine by hormones

In rat small intestine, also short-term regulation of Sglt1 by glucagon 37, glucagon-like peptide 2 (GLP-2), cholecystokinin (CCK), prostaglandin E2, EGF, leptin, insulin, and resistin-like molecule beta (RELM-β) was observed [64, 67, 149, 158, 211, 261, 305, 347, 378–380]. Glucagon 37 and GLP-2 are secreted by L cells whereas CCK is secreted by small intestinal I cells. It was observed that Sglt1-mediated d-glucose uptake in the small intestine was rapidly upregulated by vascular infusion with glucagon 37 or GLP-2 whereas it was rapidly downregulated by serosal application of CCK. Receptors for glucagon 37 and CCK are located in the BLM of enterocytes whereas enterocytes do not contain a receptor for GLP-2 [35, 268, 301, 435]. GLP-2 rather binds to receptors in enteric neurons [35, 140] and stimulates neuronal activation of enterocytes. This leads to an increase of intracellular cAMP that activates AMPK, a signal for short-term upregulation of the exocytotic pathway of SGLT1/Sglt1 (Fig. 3). RS1 is probably involved in the GLP-2-mediated short-term regulation of SGLT1 because the effect of GLP-2 infusion was blunted when the Golgi was dissociated by brefeldin A [64]. L cells in the small intestine express the sweet taste receptor T1R1/T1R3 and secrete GLP-2 directly after application of a high concentration of d-glucose or the artificial sweetener sucralose to the mucosal side [268]. In mice in which the sweet taste receptor or the GLP-2 receptor was removed, long-term upregulation of Sglt1 on the mRNA and protein levels in response to carbohydrate-rich diet was blunted [257, 268]. It has been suggested that the sweet taste receptor is also involved in glucose-dependent short-term upregulation of Sglt1; however, no experimental evidence has been provided.

After 20-min arterial perfusion of rat jejunum with 10 nM insulin, phlorizin-inhibited glucose absorption was decreased suggesting downregulation of Sglt1 in the BBM after binding of insulin to the insulin receptor isoform B (IR-B) located in the BLM [14, 32, 305]. Employing an isolated, jointly perfused rat preparation of the small intestine and liver, it was observed that insulin in the portal vein promoted a rapid increase of phlorizin-inhibited d-glucose absorption [380]. This stimulation was supposed to be mediated via insulin binding to a portal insulin receptor that activates parasympathetic hepatoenteral nerves which upregulate Sglt1 in the BBM [380].

Short-term upregulation of SGLT1/Sglt1 was also observed after interaction of EGF and prostaglandin E2 [67, 149, 261, 347]. Activation of the prostaglandin E_2_ receptor in the BBM of rodents promoted the increase of cytosolic cAMP that stimulated the exocytotic Sglt1 pathway [85, 347, 350, 421].

In addition, short-term downregulation of SGLT1/Sglt1 abundance in small intestinal BBM can be induced by leptin [95, 247]. Leptin is secreted by salivary glands and chief cells in gastric mucosa [20, 49, 139]. By binding to a protein with structural similarity to the soluble leptin receptor, leptin becomes resistant to the acidic pH in stomach [50]. In the small intestine, leptin binds to the leptin receptor in the BBM and mediates PKC-dependent downregulation of SGLT1/Sglt1 [29].

Moreover, RELM-β appears to be involved in short-term upregulation of SGLT1/Sglt1 [211]. RELM-β is expressed in the digestive tract where it plays a role in host defense but it is also observed in the blood and can act as hormone [16, 278, 357, 372]. After application of 1 nM RELM-β to the mucosal side of rat small intestine, RELM-β decreased glucose-induced short-circuit currents and Sglt1 abundance in the BBM within 2 min [211].

#### Long-term regulation in the small intestine in response to carbohydrate-rich diet

Enhancement of Sglt1 expression and capacity for glucose absorption in response to increased dietary carbohydrates was observed in herbivores and omnivores but not in carnivores [37, 43, 86, 100, 257, 354, 362]. In rodents, d-glucose, d-fructose, d-galactose, AMG, 3-O-methyl d-glucoside (3-OMG), d-mannose, d-xylose, and/or different artificial sweeteners were shown to increase Sglt1 expression on mRNA and/or protein level; however, I has not been clarified whether the different compounds address the same regulatory mechanism [28, 199, 233, 257, 265, 362]. After 5-day application of diets containing high amounts of d-glucose, AMG, d-galactose, d-fructose, d-mannose, or d-xylose to rats, Sglt1 mRNA in the small intestine was increased [265]. Monosaccharide-dependent upregulation of Sglt1 mRNA may occur relatively rapidly. Thus, an increase of Sglt1 mRNA was also observed in the small intestine of rats that had been fed for 12 h with diets containing high amounts of d-glucose, d-fructose, or sucrose [199, 430]. The data indicate that upregulation of SGLT1 in response to dietary monosaccharides occurs on the level of mRNA; however, additional mechanisms including changes in metabolism are involved (see below).

Measuring phlorizin binding to mouse enterocytes from different regions of villi and crypts at different time intervals after switching to carbohydrate-rich diet, it was observed that carbohydrate-mediated induction of Sglt1 expression occurred within the crypts [110, 111, 113]. Whereas phlorizin binding in the crypts was increased 12 h after exposure to carbohydrates, 3 days were required until phlorizin binding on the tips of the villi was maximally increased. This time lag is supposed to be due to the time required for crypt enterocytes to migrate onto villi. Experiments with mice in which the taste receptor T1R3 or the G protein α-gustin that are expressed in G cells, were removed revealed that long-term upregulation of Sglt1 mRNA and Sglt1 protein in mice by glucose and artificial sweeteners was dependent on taste reception [257, 268]. Because mucosal application of d-glucose or sucralose leads to an acute secretion of GLP-2 and enteric nerves contain GLP-2 receptors (see below), taste receptor–mediated GLP-2 release from G cells and neuronal stimulation of Sglt1 transcription are supposed to be critically involved in diet-dependent upregulation of Sglt1 expression in rodents.

RELM-β which is present in the blood is involved in long-term regulation of Sglt1 and Glut2 in response to saturated free fatty acids and glucose [120, 129, 372]. In response to high glucose, the concentration of RELM-β in rat small intestinal enterocytes was decreased which resulted in a long-term decrease and increase of Sglt1 and Glut2 in the BBM, respectively [120, 211]. These inverse changes may represent a mechanism for energy conservation during chronic high glucose load (see below).

Ruminant sheep exhibit an extensive, carbohydrate-dependent regulation of SGLT1. In the small intestine of lambs, SGLT1 is highly expressed during breastfeeding when d-glucose and d-galactose enter the small intestine whereas SGLT1 expression is largely reduced after weaning when the rumen where carbohydrates are fermented has maturated and no hexoses enter the small intestine. In adult sheep, SGLT1 protein in the BBM and SGLT1-mediated glucose uptake in BBM vesicles was decreased > 200-fold whereas SGLT1 mRNA was only decreased about 4-fold [233, 355]. When the small intestine of adult lambs was perfused with 10 mM d-glucose, SGLT1 protein in the BBM and SGLT1-mediated glucose transport into BBM vesicles were increased > 40-fold and SGLT1 mRNA was increased about 2-fold. After perfusion with 10 mM mannitol, small intestinal expression of SGLT1 was not changed. Some upregulation of SGLT1 was also observed after perfusion with the membrane impermeable d-glucose analogue, di(glucos-6-yl)poly(ethylene glycol) 600 that does not interact with SGLT1 [99]. The data indicate a dramatic upregulation of SGLT1 in the presence of d-glucose in the diet that occurs partially on the level of mRNA but mainly on the translational or post-translational level. The upregulation appears to be partially independent of glucose uptake and to be partially mediated via sweet taste receptors.

### Regulation of GLUT2

#### Basic knowledge about transcriptional regulation in human

**Fig. 5:**
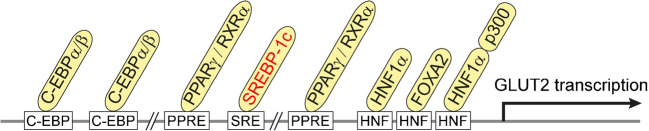
Transcriptional regulation of GLUT2. Response elements in the promotor of human GLUT2 and transcription factors that are supposed to be involved in the regulation of GLUT2/Glut2 in the small intestine are indicated. SREBP-1c participates in d-glucose-dependent regulation of Glut2. C-EBP, CCAAT enhancer–binding protein; PPAR, peroxisome proliferator-activated receptor; RXR, retinoid X receptor; SREBP, sterol receptor element–binding protein; HNF, hepatic nuclear factor; FOX, forkhead box; p300, histone acetyltransferase p300

Various transcription factors that interact with consensus sequences in the promotor region of human GLUT2 and influence transcription in hepatocytes have been identified (Fig. 5) [7]. CCAAT/enhancer-binding proteins (C/EBP) α and β form dimers which upregulate GLUT2 transcription [194]. Peroxisome proliferator–activated receptor (PPAR) γ that dimerizes with the retinoid X receptor (RXR) α is involved in the antidiabetic action of thiazolidinedione that binds to PPARγ [281]. In hepatocytes, also transcriptional upregulation of GLUT2 by hepatic nuclear factors has been observed and interaction of HNF1α and forkhead box (FOX) A2 with HNF consensus sequences in the GLUT2 promoter has been demonstrated [7, 60]. HNF1α is most abundantly expressed in the liver but has been also detected in the intestine, kidney, and spleen [26]. Importantly, glucose uptake and transcription of GLUT2 in hepatocytes are stimulated by glucose, fructose, and sorbitol but not by non-metabolized 2-DOG or 3-OMG [17, 318]. Data have been presented showing that the transcription factor sterol regulatory element-binding protein (SREBP) 1c that is also expressed in the intestine, is involved in this regulation [38, 114, 173, 397].

#### Diurnal regulation in the small intestine

Expression of Glut2 mRNA in the small intestine undergoes diurnal circuity that is coordinated with Sglt1 and Glut5. In rodents that were fed ad libitum and kept with a 12-h light/dark cycle, expression of Glut2 in the small intestine peaked at the end of the light phase and was lowest at the end of the dark phase [69, 108, 177, 390]. Vagal innervation was shown to be critically involved [390] and evidence was presented that the transcription factor BMAL1 that regulates central clock genes is required [177**].**

#### Short-term post-translational regulation in the small intestine by glucose

In response to small intestinal glucose concentrations of 30 mM or above, Glut2 is rapidly inserted into the BBM of the enterocytes [132, 134, 152, 189, 192]. This enables an effective, energy-saving d-glucose uptake at glucose concentrations far above the *K*_m_ of SGLT1. In parallel, the capacity for d-fructose uptake into the enterocytes is increased. When high luminal glucose dissipates, Glut2 abundance in the BBM decreases with a delay of minutes [189, 396]. Evidence has been presented that the glucose-dependent increase of Glut2 in the BBM is due to upregulation of exocytosis of Glut2-containing vesicles; however, it has not been elucidated whether the vesicles originate from the BLM, an intracellular vesicle pool or the Golgi.

**Fig. 6:**
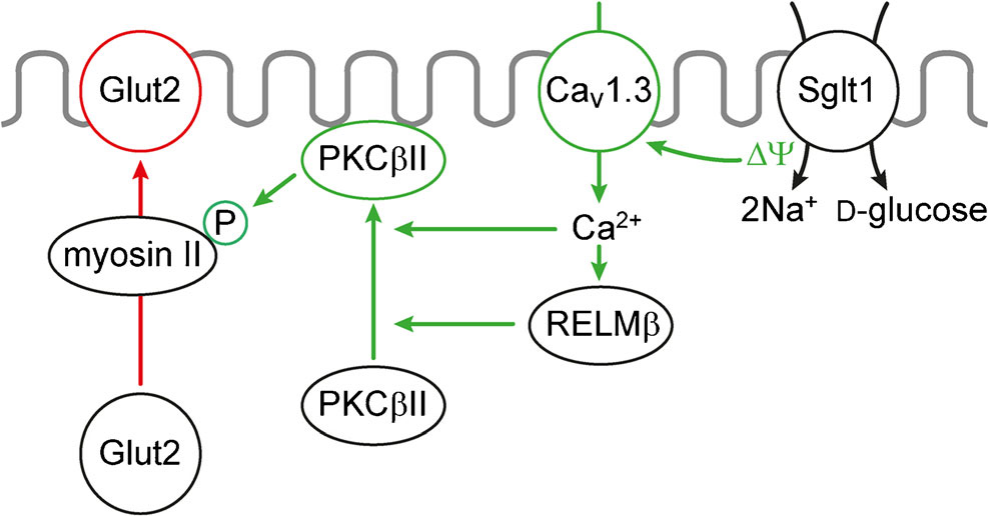
Model depicting components that are involved in targeting of Glut2 to the BBM at high luminal glucose concentrations. The underlying experiments were performed in rats. ∆Ψ, membrane depolarization due to Na^+^-d-glucose cotransport; Ca_v_, voltage-dependent Ca^2+^channel; RELM, resistin-like molecule

The mechanism of glucose-dependent incorporation of Glut2 into the BBM has been investigated in some detail. PKC may be involved because the BBM abundance of Glut2 was also increased rapidly when the small intestine was incubated with PMA [5, 152]. Maximal electrogenic glucose transport by Sglt1 at high intestinal glucose when Sglt1 in the BBM is upregulated and the transporter operates at *V*_max_ is supposed to trigger Glut2 incorporation into the BBM (Fig. 6). Sglt1-mediated Na^+^-d-cotransport causes a depolarization of the BBM that activates Ca^2+^ uptake via Ca^2+^ channel Ca_v_1.3. [269, 270]. The increased intracellular Ca^2+^ stimulates phosphorylation of myosin II by protein kinase C βII (PKC βII) that is activated upon recruitment to the plasma membrane [192, 250, 251, 401]. Recruitment of PKC βII to the plasma membrane is also promoted by RELM-β that has been also shown to increase Glut2 in the BBM [211]. Phosphorylation of myosin II leads to a rearrangement of the subapical terminal web that is supposed to facilitate BBM insertion of Glut2. Because Ca^2+^ uptake into enterocytes was stimulated maximally at 20 mM d-glucose but higher glucose concentrations were required for insertion of Glut2 into the BBM, an additional glucose-dependent process has been postulated [192]. This process could be related to glucose-dependent phosphorylation of AMPK or to paracrine effects of taste receptor activation in enteroendocrine cells [192, 211, 412].

#### Short-term regulation in the small intestine by insulin and GLP-2

Short-term effects of insulin on plasma membrane abundance of Glut2 were investigated in the small intestine of mice that had been kept for 5, 15, or 30 days on fructose-rich diet [396]. After 15 and 30 days on fructose-rich diet, the abundance of Glut2 in the BBM was increased due to long-term upregulation (see below), and the animals became resistant to insulin after 30 days on fructose-rich diet. When the insulin-sensitive animals kept for 15 days on fructose-rich diet were infused with insulin under hyperinsulinemic-euglycemic clamp conditions, the increased BBM abundance of Glut2 due to the fructose-rich diet was decreased whereas this effect was not observed in the insulin-resistant animals. The effect of insulin on short-term, glucose-induced trafficking of Glut2 to the BBM was investigated in the mice that had been fed for 5 days with fructose-rich diet. Thirty minutes after glucose gavage of these mice, Glut2 in the BBM was largely increased; however, this increase was blunted when insulin had been injected prior to glucose gavage. The data indicate that insulin induces short-term removal of Glut2 from the BBM and prevents short-term glucose-dependent trafficking of Glut2 to the BBM.

Trafficking of Glut2 to the BBM combined with an increase of glucose absorption in rat small intestine was observed after 1-h vascular perfusion with GLP-2 [18]. Evidence was presented that the GLP-2-induced upregulation of Glut2 in the BBM involves activation of enteric neurons [268].

#### Long-term carbohydrate-dependent regulation in the small intestine

After 5-day application of diets containing high amounts of d-glucose, d-galactose, or d-fructose, the abundance of Glut2 mRNA in rat enterocytes was increased whereas high amounts of AMG, d-mannose, or d-xylose were not effective [265]. Noteworthy, post-translational upregulation of Glut2 and Glut5 was observed in mice after 25-week application of high-fructose diet [91]. This fructose-dependent upregulation was associated with mRNA upregulation of thioredoxin-interacting protein (TXNIP) that is involved in regulation of various metabolic pathways [300]. TXNIP was shown to bind to human GLUT2 and GLUT5 and increased the abundance of these transporters after coexpression in Caco-2 cells [91]. In nondiabetic rodents on standard chow, Glut2 was almost exclusively located in the BLM of enterocytes between meals. At variance, in animals receiving a glucose-rich and/or fructose-rich diet, Glut2 was located in both the BLM and the BBM, between meals [28, 134, 396].

### Regulation of GLUT5

#### Knowledge about transcriptional and epigenetic regulation

The promoter regions of human, rat, and/or mouse GLUT5/Glut5 contain response elements for cAMP (CRE), glucocorticoid receptor (GR), liver X receptor (LXR), carbohydrate-responsive element-binding protein (ChREBP), and thyroid hormone receptor/retinoid X receptor heteromer [255, 260, 283, 385, 441].

In Caco-2 cells transfected with reporter gene constructs containing the human GLUT5, promoter expression was stimulated by adenylate cyclase [255]. Using the same experimental setup, transcriptional regulation by thyroid hormone was observed [260]. It was shown that binding of thyroid hormone receptor/retinoid X receptor heteromers to the promotor was blunted by thyroid hormone.

In rat small intestine, Glut5 mRNA was upregulated after weaning provided that systemic glucocorticoids were present and fructose had been ingested [385]. Epigenetic regulation is probably involved because the glucocorticoid-promoted upregulation of Glut5 transcription after weaning was associated with histone H3 acetylation of the promotor [385]. At variance, sucrose-dependent upregulation of Glut5 mRNA observed after feeding of starved rats with sucrose-rich food was associated with histone H3 acetylation of the encoding DNA sequence [162]. Functionality of liver X receptor α response element (LXRE) in the murine Glut5 promoter was suggested by the observation that Glut5 mRNA was increased by a LXR agonist [441]. ChREBP and thyroid hormone are probably involved in upregulation of Glut5 transcription by d-fructose. Whereas in wild-type mice on high-fructose diet the abundance of Glut5 mRNA in the small intestine was eight times higher compared to standard diet, fructose in the diet had no effect on small intestinal abundance of Glut5 mRNA in ChREBP knockout mice [283].

The abundance of GLUT5 mRNA is also regulated on the level of mRNA stability. Thus, the degradation of GLUT5 mRNA in Caco-2 cells was slowed down by cAMP and fructose [135, 255]. This increase of message stability was due to cAMP-dependent binding of cytosolic proteins to untranslated regions of GLUT5 mRNA [135].

#### Diurnal regulation in the small intestine

In rats kept with a 12-h light/dark cycle and free access to food, Glut5 mRNA in the jejunum was increased in the late light/early dark phase similar to the mRNAs of Glut2 and Sglt1 [58, 69, 108, 177, 390].

#### Fructose-dependent upregulation in the small intestine

So far, no short-term post-translational upregulation of GLUT5/Glut5 by monosaccharides has been described. However, Glut5 in the BBM of rat small intestine was increased within minutes after application of inhibitors of signal pathways involving epithelial receptor kinase (ERK)/mitogen-activated protein (MAP) kinase or phosphoinositol-3 kinase [151].

In rodents, upregulation of Glut5 on the level of mRNA was observed in response to dietary d-fructose whereas other monosaccharides were not effective. For example, an increase of small intestinal Glut5 mRNA was observed when rats were fed for 5–7 days with a d-fructose-rich diet whereas diets enriched with d-glucose, d-galactose, AMG, d-mannose, or d-xylose were not effective [45, 265]. When mice on a carbohydrate-poor diet were switched to a d-fructose-rich diet, transcription of Glut5 was enhanced 12 h later [135, 199]. The onset of dietary upregulation of Glut5 mRNA only requires several hours. In mice, 4-h perfusion of small intestine with buffer containing 100 mM fructose provoked a significant increase of Glut5 mRNA whereas perfusion with 100 mM glucose was not effective [77, 385]. In addition to mRNA, also Glut5 protein abundance in cytosol and BBM were increased.

### Carbohydrate-dependent regulation of monosaccharide absorption

**Fig. 7:**
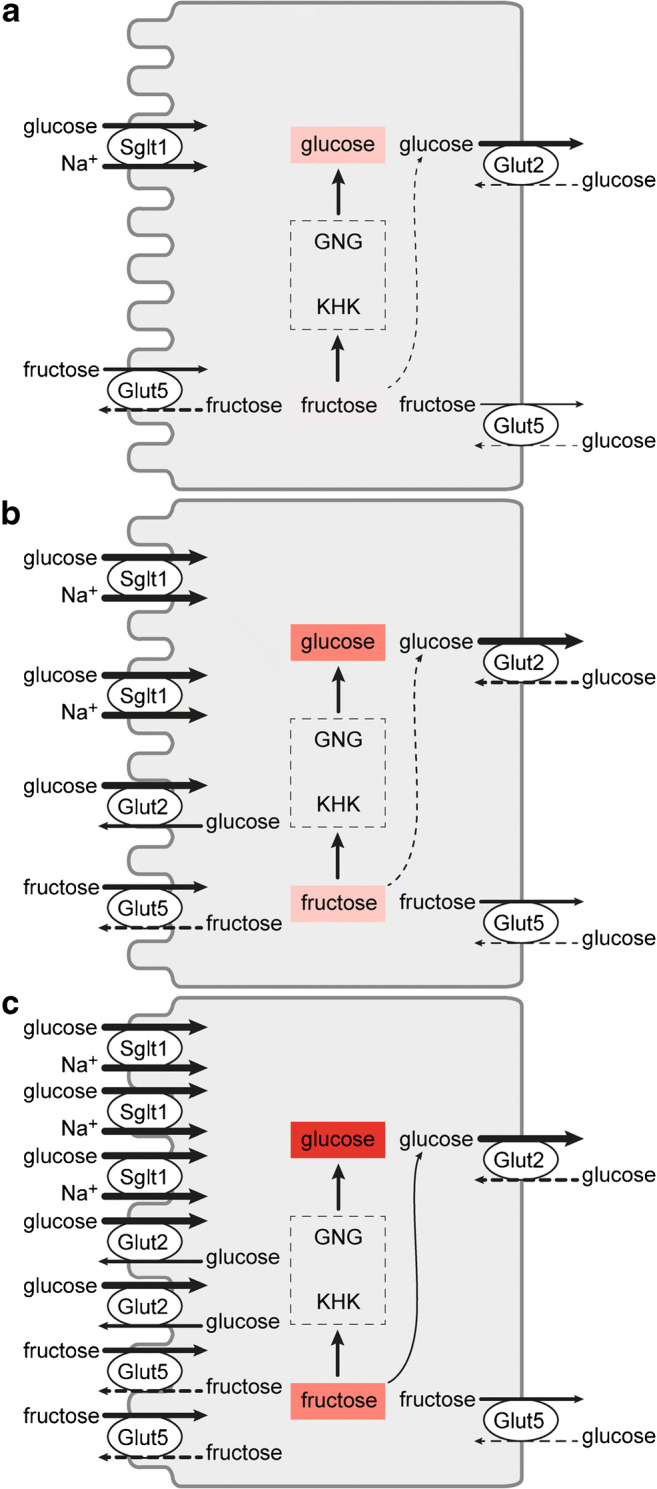
Plasma membrane localization and abundance of Sglt1, Glut2, and Glut3 in response to carbohydrates in the diet after ingestion of a carbohydrate-poor or a carbohydrate- and sucrose-rich meal. The underlying experiments were performed in rats. **a** Carbohydrate-poor diet after a carbohydrate-poor meal. **b** Carbohydrate-poor diet after a sucrose-rich meal. **c** Carbohydrate-rich diet after a sucrose-rich meal. KHK, ketohexokinase; GNG, gluconeogenesis

In the following, it will be discussed how the above-described monosaccharide-dependent regulations of Sglt1, Glut2, and Glut5 affect small intestinal absorption of d-glucose and d-fructose. Three regulatory states that are fundamentally different from each other will be considered: first, the situation in individuals on carbohydrate-poor (low-carb) diets after low-carb meals (Fig. 7a); second, the situation in individuals on low-carb diets after carbohydrate-rich (high-carb) meals containing large amounts of sucrose that is rapidly split into d-glucose and d-fructose by sucrase-isomaltase (Fig. 7b); and third, the situation in individuals on a high-carb diets after meals containing large amounts of sucrose (Fig. 7c).

**Fig. 8:**
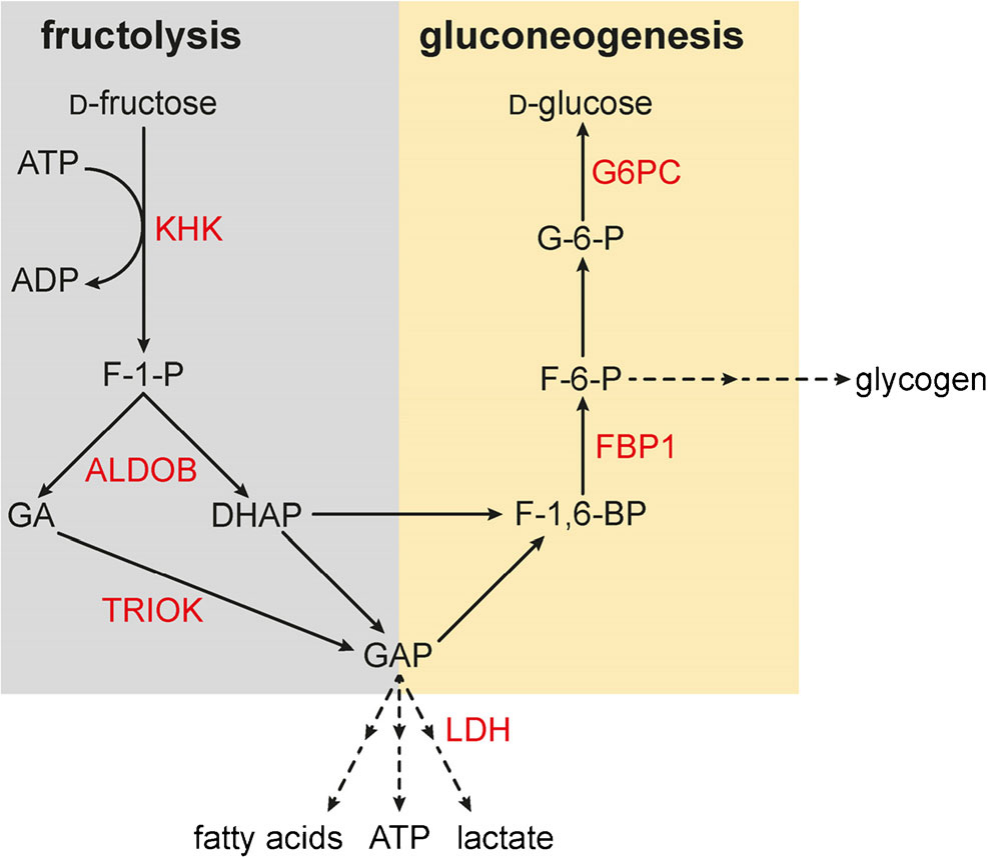
Fructolysis and gluconeogenesis, and effects of removal of carbohydrate-responsive element (ChoRE)–binding protein in mice on fructose-dependent expression of the involved enzymes. Enzymes that are upregulated by high-fructose diet in the presence but not in the absence of ChoRE are indicated in red. Of note, also Glut5 in the luminal membrane of enterocytes mediating d-fructose uptake is only upregulated by high-fructose diet if ChoRE-binding protein is expressed in the enterocytes. Ketohexokinase (KHK), aldolase B (ALDOB), triosekinase (TRIOK), glucose-6-phosphatase (G6PC), fructose-1,6-biphosphatase (FBP1), and lactate dehydrogenase (LDH) are upregulated

In the small intestine of rats on low-carb diet after ingestion of low-carb food, when the concentrations of d-glucose and d-galactose are below the respective *K*_m_ values of SGLT1/Sglt1-mediated uptake (Table 1), the abundance of Sglt1 and Glut5 in the BBM of the enterocytes and of Glut2 in the BLM is low and Glut2 is not present in the BBM (Fig. 7a). Under this condition, small concentrations of d-glucose and d-galactose after low-carb meals are effectively absorbed by secondary active transport by Sglt1 across the BBM followed by passive diffusion of intracellularly enriched monosaccharides across the BLM via Glut2. d-Fructose enters the enterocytes via Glut5 in the BBM. In a recent study performed in mice, evidence has been provided that about 90% of d-fructose entering the enterocytes is metabolized and increases the intracellular pool of d-glucose due to gluconeogenesis [179]. Hence, the intracellular d-fructose concentration is low and only small amounts of d-fructose leave the cells via Glut2 and Glut5 (Fig. 7). In the small intestine, ketohexokinase (KHK) phosphorylates d-fructose in position 1 and provides the starting compound for fructolysis (Fig. 8). After removal of KHK in mice, d-fructose in the serum after high-fructose feeding was largely increased whereas fructose-induced hyperglycemia was blunted [296]. Catalyzed by aldolase B (ALDOB), d-fructose-1-phosphate is split into glyceraldehyde (GA) and dihydroxyacetone phosphate (DHAP). DHAP and glyceraldehydephosphate (GAP) formed by triosekinase (TRIOK)-mediated phosphorylation of GA enter gluconeogenesis (GNG) (Fig. 8). GAP enters other metabolic pathways including fatty acid synthesis.

Glut2 located in the BLM that transports d-glucose, d-galactose, and d-fructose, is probably not the only transporter for d-glucose export across the BLM because glucose absorption was not significantly decreased in Glut2 knockout mice [381]. Because it was observed that d-glucose absorption in Glut2 knockout mice was blocked when glucose-6-phosphate (G-6-P) translocase in the endoplasmic reticulum (ER) was inhibited, the hypothesis was raised that uptake of glucose-6-phosohate into the ER and exocytosis is involved in glucose transport across the BLM [381]. So far, unequivocal experimental evidence for the existence of this translocation pathway is missing. Measurement of glucose absorption in the absence and presence of inhibitors of exocytosis and uptake measurements in vesicles from small intestinal BLMs of Glut2 knockout mice are recommended. It is possible that Glut1, Glut7, Glut8, or Glut12 is targeted to the BLM when Glut2 is removed and provides a compensatory path for efflux of d-glucose.

When rats on low-carb high diet had ingested high-carb meals containing large amounts of sucrose, it was observed that the amount of Sglt1 in the BBM was increased and that Glut2 was present not only in the BLM but also in the BBM (Fig. 7b). After meals containing large amounts of sucrose, the concentrations of d-glucose and d-fructose in the small intestinal lumen close to the BBM are > 100 mM because sucrose-isomaltase degrades sucrose effectively. Under these conditions, Sglt1 and Glut2 transport d-glucose and Glut5 transports d-fructose at *V*_max_ across the BBM whereas Glut2 transports d-fructose with about half maximal efficacy (Table 1). d-Glucose uptake across the BBM is mediated about one-third by Sglt1 and two-thirds by glut2 [192], whereas d-fructose uptake across the BBM is mediated to similar proportions by Glut5 and Glut2 [134]. Computer simulation studies on measurements performed in Caco-2 cells support the concept that facilitative diffusion of d-glucose across the BBM contributes to d-glucose uptake into enterocytes at high glucose concentrations [6]. The bulk of intracellular d-fructose at high-carb diet is phosphorylated, split, and transformed to d-glucose by GNG. Transport of the relatively high amounts of intracellular d-glucose across the BLM is supposed to be mediated by GLUT2/Glut2, whereas transport of the relatively small amounts of intracellular d-fructose is supposed to be mediated by GLUT5/Glut5 and GLUT2/Glut2.

It has been observed that small intestinal absorption of d-fructose was stimulated by high concentrations of luminal d-glucose [159, 330–332]. In humans, a similar effect of d-glucose was observed on the absorption of sorbitol that is not transported by GLUT5 [331], and d-fructose absorption was also stimulated by amino acids which are translocated by sodium-amino acid cotransporters across the BBM [159]. The stimulation of d-fructose by luminal d-glucose or amino acids is probably due to water removal from the intestinal lumen that leads to an increase of the luminal fructose concentration and enhances transport efficacy of GLUT5/Glut5. Cotransport of d-glucose and amino acids with sodium leads to an increase of sodium in the intercellular spaces and promotes permeation water across the tight junctions.

In response to high-carb diet containing large amounts of sucrose, the overall expression of Sglt1, Glut2, and Glut5 in the enterocytes is increased (Fig. 7c). Between meals and after uptake of low-carb meals, Sglt1 and Glut5 are located in the BBM whereas Glut2 and probably also Glut5 are located in the BLM as depicted in Fig. 7 b. After ingestion of high-carb food with high amounts of sucrose, the same short-term regulation takes place as in individuals on low-carb diet. Thus, additional molecules of Sglt1 are incorporated into the BBM and additional amounts of Glut2 and Glut5 are targeted to the BBM (Fig. 7c). In consequence, the capacity for absorption of d-glucose and d-galactose is maximized leading to maximal increases of plasma glucose after sucrose ingestion. Also, the capacity of d-fructose uptake into the enterocytes is maximized leading to maximal d-glucose generation by GNG. Under these conditions, there is a maximal risk for fructose intolerance (see below).

## Expression and function of SGLT1 and GLUTs in enteroendocrine cells

### Secretion and functions of enterohormones

Secretion of the enterohormones GLP-1, glucagon-like peptide 2 (GLP-2), and peptide YY (PYY) by L cells, GIP by K cells, CCK by I cells, neurotensin by N cells, and serotonin by EC cells is involved in the regulation of appetite, gastric emptying, small intestinal motions, d-glucose absorption, and d-glucose metabolism [21, 62, 137, 314, 317, 320]. The effects of GLP-2, ghrelin, CCK, neurotensin, and serotonin on appetite and functions of the alimentary tract are mediated by stimulation of neuronal receptors in peripheral neurons and/or central neurons [24, 314, 369]. At variance, effects of GLP-1 and GIP on d-glucose metabolism are mainly mediated via stimulation of insulin secretion by β cells in pancreatic islets and of insulin-independent d-glucose disposal [21, 78, 210]. In addition to amino acids, peptides, and fatty acids, secretion of GLP-1 by L cells and of GIP by K cells is stimulated by d-glucose and d-fructose [72, 193, 215, 251]. In rodents and humans, L cells are mainly expressed in the ileum and colon whereas K cells are mainly expressed in the jejunum [317, 320]. Some L cells are also expressed in the duodenum [143, 382, 393].

### Roles of glucose transporters for secretion of GLP-1 and GIP

Discussing the role of glucose transporters in d-glucose sensing by small intestinal L and K cells, the limitations of the available data must be considered. First, our knowledge about the mechanisms involved in d-glucose-dependent stimulation of GLP-1 and GIP secretion is derived from studies in different species and in cultured cells. Second, different populations of the enteroendocrine cells (EECs) may have been investigated because L and K cells consist of different cell populations that secrete partially different enterohormones [101, 143, 384, 386]. Third, the functional properties of EECs may have been changed in response to nutrient exposition and during diabetes [15, 52, 345, 382, 393].

The available data indicate that SGLT1/Sglt1 and GLUT2/Glut2 are critically involved in glucose-dependent stimulation of GLP-1 and GIP secretion in the small intestine at high luminal d-glucose concentrations [132, 271, 322, 382], and that activation of the sweet taste receptor T1R2/T1R3 heterodimer may participate [121, 178]. Additional proteins have been associated with d-glucose-dependent secretion of GLP-1 and GIP. These are (a) voltage-dependent Ca^2+^ channel(s) (Ca_v_) and the ATP-regulated K^+^ channel Kir6.2/Sur1 (K_ATP_) [251, 279, 282, 315, 325, 382, 393]. Sglt1-mediated d-glucose uptake triggers glucose-dependent secretion of GLP-1 and GIP at low and high luminal d-glucose concentrations. Cotransport of sodium and d-glucose by Sglt1 leads to depolarization of the luminal plasma membrane and induces Ca^2+^ uptake via (a) voltage-dependent Ca^2+^ channel(s) [295, 316]. Ca^2+^ uptake may stimulate insertion of Glut2 into the luminal membrane in enterocytes (Fig. 6). At luminal d-glucose concentrations far above the *K*_m_ for SGLT1/Sglt1, GLUT2/Glut2 in the luminal membrane is supposed to mediate a considerable fraction of d-glucose uptake in addition to Sglt1. High intracellular d-glucose increases carbohydrate metabolism [294, 316, 393]. The increased metabolism results in an increase of intracellular ATP that may lead to closure of K_ATP_ channels and promote depolarization of the plasma membrane. This may result in the opening of Ca_v_ channels located in the basolateral membrane [316]. The increase of intracellular Ca^2+^ may be further enhanced by Ca^2+^-promoted Ca^2+^ release from the endoplasmic reticulum. The increase of intracellular Ca^2+^ causes the exocytosis of vesicles containing GIP and GLP-1.

Experimental evidence for a pivotal role of Sglt1 in glucose-induced secretion of GLP-1 and GIP was provided as follows. In mice, the early secretion of GLP-1 after gavage with d-glucose was blunted and the secretion of GIP was abolished when SGLT1 was removed [132, 295, 322]. Perfusing rat small intestine in vivo, it was observed that the secretion of GIP and/or GLP-1 was stimulated when the luminal d-glucose concentration was increased from 5 to 100 mM d-glucose and that this increase was blunted by 45% when Sglt1 was inhibited with phlorizin [251]. In humans, the secretion of GIP and/or GLP-1 during oral glucose tolerance tests (OGTTs) was decreased when SGLT1 was inhibited by oral application of an SGLT1/Sglt1 inhibitor [88, 176]. It was observed in rodents and humans that secretion of GLP-1 and GIP was also stimulated by non-metabolizable sugars such as AMG and 3-OMG [251, 271, 321, 382, 425]. This indicates that metabolism and ATP-mediated closure of the K_ATP_ channel were not critical under the employed conditions. However, in one study performed in humans, the stimulation of GLP-1 secretion by 300 mM d-glucose was more pronounced than the stimulation by 300 mM AMG [382].

Relevance of GLUT2/Glut2 for stimulation of GLP-1 and/or GIP by very high glucose concentrations in the intestinal lumen was indicated by the following data. In experiments in which rat small intestine was perfused, secretion of GLP-1 and GIP was stimulated by 100 mM d-glucose in the presence of phlorizin, and this Sglt1-independent stimulation was blocked when Glut2 was inhibited with phloretin or cytochalasin B [251]. In the same study, it was observed that the secretion of GLP-1 and GIP in the presence of 100 mM d-glucose was decreased when K_ATP_ channels were blocked by tolbutamide. In another study in rat, no stimulation of GLP-1 secretion by 1.1 M luminal d-glucose was observed when the K_ATP_ channels were opened by diazoxide or when the Ca_v_ channels were blocked by veratridine [215]. After removal of Glut2 in mice, the increase of GLP-1 in the blood during the OGTT was blunted whereas the increase of GIP was not changed [51]. The relevance of GLUT2/Glut2-mediated glucose uptake for GLP-1 secretion was approved for humans. d-Glucose-dependent GLP-1 secretion by isolated mucosa of healthy individuals was affected by phloretin, the ATP synthesis inhibitor 2,4 dinitrophenol, the K_ATP_ channel blocker tolbutamide, and the L-type Ca^2+^ channel blocker nifedipine [382]. At variance, in patients with type 2 diabetes, no effects of the K_ATP_ channel inhibitors glibenclamide and repaglinide on secretion of GLP-1 and GIP during OGTTs were observed [371].

In rats and humans, secretion of GLP-1 was also observed after luminal application of d-fructose [321, 370]. Because fructose is transported by the facilitated diffusion transporters GLUT5/Glut5 and GLUT2/Glut2 but not by SGLT1*/*Sglt1, GLP-1 secretion is most probably not triggered by transport-related depolarization of the luminal membrane. d-Fructose-induced secretion is probably mediated by effects of intracellular d-fructose on metabolism leading to increased intracellular ATP.

The sweet taste receptor T1R2/T1R3 is supposed to be involved in secretion of GLP-1 by high d-glucose concentrations. It may also mediate secretion of GIP and GLP-1 by artificial sweeteners; however, conflicting data concerning this function have been reported. In humans, GLP-1 secretion after gastrical or duodenal application of a d-glucose bolus was impaired by sweet taste receptor inhibitor lactisole [128]. In mice, the stimulation of GLP-1 secretion after gavage with d-glucose was blunted when the sweet taste receptor component T1R3 or the G protein subunit α-transducin had been removed [178]. In perfused rat small intestine, the secretion of GIP and/or GLP-1 was(were) increased by luminal application of artificial sweeteners [215, 251]. In contrast, no stimulation of GLP-1 secretion by artificial sweeteners was observed in Zucker diabetic fatty rats and in healthy humans [121, 249, 370, 425]. Also, in mice, no increase of plasma GIP was observed 15 min after gavage with the artificial sweetener saccharin [282].

### Roles of glucose transporters for secretion of neurotensin

Neurotensin is produced by endocrine cells in intestine with low expression in the duodenum, caecum, and colon; intermediate expression in the jejunum; and high expression in the ileum [55, 214, 320]. EECs expressing neurotensin have been designated as N cells [320]; however, since coexpression of GLP-1, GIP, or CCK with neurotensin has been observed [101, 143, 386], N cells can be considered subpopulations of L, K, or I cells. Neurotensin induces small intestinal muscle contractions, promotes arterial hypotension, and increases plasma d-glucose by affecting hepatic carbohydrate metabolism [56, 119]. Stimulation of small intestinal secretion of neurotensin by luminal d-glucose was demonstrated in rat and human [79, 213, 214]. In rat, evidence was provided that Sglt1 and Glut2 are involved in glucose sensing and that d-glucose metabolism, K_ATP_ channels, and Ca_v_ channels may participate. It was observed that perfusion of rat small intestine with 5 mM and 250 mM d-glucose resulted in 4.4- and 12-fold stimulation of neurotensin secretion [79]. The stimulation at both glucose concentrations was prevented by luminal application of phlorizin whereas it was not changed when phloretin was applied from the basal cell side. These observations suggested a pivotal role of SGLT1/Sglt1 for neurotensin secretion at low and high luminal d-glucose concentrations. Similar to d-glucose-induced stimulation of GLP-1 secretion by L cells, d-glucose-induced stimulation of neurotensin secretion is supposed to be due to Sglt1-mediated d-glucose transport plus Sglt1 promoted incorporation of Glut2 into the luminal membrane that becomes relevant at high glucose concentrations. An increase of plasma neurotensin after gavage with d-glucose was also observed in humans [213]. Using perfused rat small intestine, the mechanism how high luminal d-glucose concentrations stimulate the secretion of neurotensin was investigated [214]. By luminal application of 1.1 M d-glucose neurotensin secretion was stimulated whereas vascular application of d-glucose was not effective. d-Glucose-mediated stimulation of neurotensin secretion was totally abolished in the presence of luminal phlorizin and was decreased by about 90% in the presence of luminal phloretin. Stimulation of neurotensin secretion by luminal d-glucose was also abolished when ATP synthesis had been blocked with 2-4-dinitrophenol, when K_ATP_ channels had been opened by diazoxide, or when Ca_v_ channels had been blocked with veratridine. Luminal application of artificial sweeteners did not induce neurotensin secretion. The data indicate that d-glucose-mediated stimulation of neurotensin secretion by EEC cells is similar to GLP-1 secretion by L cells.

## Diseases caused by or associated with malfunctions of glucose transporters in the small intestine

### Glucose-galactose malabsorption

Glucose-galactose malabsorption (GGM) is a rare congenital autosomal recessive disease with severe neonatal diarrhea and water loss due to the inability of intestinal d-glucose and d-galactose absorption. Without therapy, the outcome is fatal. All symptoms and pathophysiological consequences of GGM can be avoided by d-glucose and d-galactose-free diets that may contain d-fructose, allowing a normal life without GGM-related health problems. GGM has been first described in 1962 [225, 242]. About one decade later, the underlying defect in GGM was attributed to defective Na^+^-d-glucose cotransport in the small intestinal BBM and recessive inheritance was detected [103, 262, 376]. In 1991, Wright and coworkers provided evidence that genetic loss-of-function single nucleotide variations (SNVs) in both DNA strands of the *SLC5A1* cause GGM whereas heterozygous carriers have no clinical symptoms [400]. Meanwhile, the *SLC5A1* genes of more than hundred GGM patients have been sequenced [187, 258, 423, 424]. With few exceptions, SNVs have been observed in *SLC5A1*. They cause missense, nonsense, frame shift and splice site mutations, and mutations in the promotor. Most missense mutations result in defects of SGLT1 trafficking to the plasma membrane whereas some cause loss of transport function [187, 258, 424]. The nonsense, frame shift, and splice site mutations lead to truncated protein whereas the mutations in the promoter induce decreased transcription.

The few cases in which GGM patients did not contain SNVs in *SLC5A1* could be due to genetic defects in proteins that are selectively involved in targeting of SGLT1 to the plasma membrane. Malabsorption of d-glucose and d-galactose may also be associated with more general defects in transporter expression that are associated with malabsorption of additional monosaccharides. For example, mutations in the gene encoding neurogen-3 that lead to depletion of enteroendocrine cells cause monosaccharide malabsorption [414]. The severe diarrhea observed in humans with neogenin-3 mutants could be prevented by diets that do not contain d-glucose, d-galactose, and d-fructose. The sensitivity of diarrhea to d-fructose indicates that the defect is not limited to SGLT1. A decrease of SGLT1 abundance in the BBM can be also induced by defects of proteins that are involved in sorting or trafficking. Such defect could be relatively specific for SGLT1 because sorting in the Golgi, and trafficking of plasma membrane transporters in enterocytes shows some specificity for individual transporters. For example, protein RS1 (*RSC1A1*) is critically involved in the d-glucose-dependent short-term regulation of SGLT1 in the small intestinal BBM and d-glucose absorption was increased in RS1 knockout mice [286].

The concept that loss-of-function SNVs in *SLC5A1* are the main cause for GGM was verified by removal of Sglt1 in mice [132]. In Sglt1 knockout mice, small intestinal glucose absorption was reduced by more than 95%. After birth and in the preweaning period, SGLT1 knockout mice appeared to be healthy at variance to newborn humans; however, they developed severe diarrhea and died within 2 weeks when they were kept on standard diet after weaning. As observed in humans with GGM, diarrhea disappeared and the Sglt1 knockout mice developed well when they were fed with a d-glucose- and d-galactose-free diet. The difference concerning neonatal diarrhea in humans with GGM compared to Sglt1 knockout mice is supposed to be due the expression of Sglt3b in mice. This rodent-specific Sglt subtype is located in the small intestinal BBM and is able to mediate phlorizin-inhibitable d-glucose uptake [10, 132].

In several newborn humans with GGM, nephrocalcinosis and nephrolithiasis were the diagnosis [1, 2, 102, 288, 365, 388]. Future studies are necessary to clarify whether nephrocalcinosis associated with GGM is due to metabolic acidosis during the diarrhea and/or to comorbidity factors that promote hypercalcemia [102, 288] and/or tubular acidification [102].

### Fanconi-Bickel syndrome

Fanconi-Bickel syndrome (FBS) is a rare congenital disease with the key symptoms hepatomegaly, nephropathy, postprandial hyperglycemia, fasting hypoglycemia, and growth retardation [106, 337]. FBS is occasionally linked with intolerance to d-glucose and d-galactose caused by impaired small intestinal carbohydrate absorption that may lead to diarrhea after ingestion of carbohydrate-rich food [337]. Sequencing the *SLC2A2* gene encoding GLUT2 in 49 patients, SNVs causing inactive or truncated transporters were detected which were homozygous in 74% of the patients [336, 338, 339]. This indicates that a defective function of GLUT2 is the predominant cause for FBS.

The impaired small intestinal absorption of d-glucose and d-galactose with unchanged absorption of d-fructose observed in some patients with FBS suffering from diarrhea is consistent with the presumed function of GLUT2 for basolateral release of monosaccharides from enterocytes and/or for GLUT2-mediated monosaccharide uptake across the BBM at very high glucose concentrations. However, in most patients, the function of GLUT2 in the small intestine is probably partially compensated by upregulation of other glucose transporters in IECs like in mice in which Glut2 was selectively removed in the small intestine [345].

### Fructose intolerance and fructose malabsorption

#### General considerations

Fructose malabsorption is one type of fructose intolerance. The umbrella term “fructose intolerance” comprises situations in which d-fructose becomes available for bacterial fermentation leading to diarrhea, flatulence, pain, and intestinal cramps [13, 27, 130, 313] and in which high d-fructose concentrations are observed in the portal vein that exhibit pathogenetic effects in the liver. d-Fructose malabsorption can be due to insufficient d-fructose uptake into enterocytes relative to the amount of d-fructose in the intestinal lumen. In addition, it can be caused by insufficient intracellular fructolysis resulting in high intracellular d-fructose concentrations that also decrease d-fructose uptake (see Fig. 7). In this case, the concentration of d-fructose in the portal vein is increased and large amounts of d-fructose may enter hepatocytes. This may lead to hepatic steatosis, nonalcoholic steatohepatitis, nonalcoholic fatty liver disease (NAFLD), and/or metabolic syndrome [241]. The incidence of fructose intolerance is correlated with the amount of sucrose and free d-fructose supplied with the food. d-Fructose in the food has been increased dramatically since the nutrients and beverages were enriched with sucrose. In 2004, male human Americans ingested on average more than 70 g of d-fructose per day in the age of 15–25 years and more than 30 g per day in the age of 1–3 years [93]. In humans, the capacity for small intestinal absorption of d-fructose is much smaller than the capacity for d-glucose absorption; it is very small after birth and increases later on in response to d-fructose in the diet [93]. In a population of healthy adults, an intestinal load of 25 g d-fructose was only absorbed completely by about one-half of the individuals [360]. However, in combination with d-glucose, the capacity for d-fructose absorption is increased due to additional fructose uptake in response to luminal water reabsorption associated with Na^+^-d-glucose cotransport [159, 399]. SNVs with impact on GLUT5 or on enzymes that are critical for fructolysis promote fructose intolerance.

#### Isolated fructose malabsorption

Individual pediatric cases of abdominal pain, colicky cramps, and diarrhea after ingestion of low amounts of d-fructose that resolve upon fructose-free diet have been assigned as isolated fructose malabsorption (IFM) [27, 411, 418]. The absence of hepatic symptoms and the occurrence of intestinal symptoms after ingestion of low amounts of d-fructose in toddlers supported the hypothesis that the observed symptoms were due to an isolated defect in absorption. Studies with mice in which Glut5 was removed indicated that GLUT5/Glut5 is pivotal for d-fructose absorption and that malfunction of GLUT5/Glut5 can induce intestinal symptoms observed in IFM [28]. In the presence of high dietary d-fructose, small intestinal fructose absorption was ~ 75% lower in adult Glut5 knockout mice compared to adult wild-type mice whereas the fructose concentration in the serum was ~ 90% lower. Seven days after receiving the high-fructose diet, the Glut5 knockout mice developed a greatly enlarged and dilated colon in contrast to the wild-type mice.

Attempts were made to determine whether mutations in GLUT5 are the main cause for fructose malabsorption observed in IFM. In one study, employing 8 patients with IFM no SNVs were detected in the coding region of *SLC2A5* [418]. In another study performed on 11 patients with diagnosed functional gastrointestinal disorder (FGID) showing fructose malabsorption and 15 healthy individuals, similar amounts of GLUT5 mRNA were observed in small intestinal biopsies [420]. The data suggest that functional defects in GLUT5 or decreased transcription of GLUT5 is not the predominant cause for fructose malabsorption in IFM and/or FGID. To exclude that posttranscriptional defects in GLUT5 expression such as impaired translation and/or trafficking cause IFM, measurements of GLUT5 protein in the BBM of small intestinal biopsies of patients with IFM must be performed. Of note, the intestinal expression of GLUT5 is very low in human newborns and increases slowly after birth in response to d-fructose in the food. Hence, a delayed upregulation of GLUT5 after birth could be one reason for IFM in toddlers [93].

#### Fructose intolerance due to genetic defects in aldolase B

Hereditary fructose intolerance (HFI) due to dysfunction of aldolase B (ALDOB) is a long known and well investigated disease [9, 61, 74, 75, 153]. ALDOB is expressed in the small intestine, liver, and kidney and catalyzes the cleavage of d-fructose-1-phosphate formed from d-fructose into d-glyceraldehyde (GA) and dihydroxyacetone phosphate (DHAP) that enters gluconeogenesis (GNG) (Fig. 8). ALDOB is regulated in a tissue-specific manner by dietary carbohydrates including d-fructose and by hormones [276]. Failure of ALDOB in enterocytes results in fructose malabsorption due to a disturbed metabolism of d-fructose leading to severe abdominal symptoms after ingestion of high amounts of d-fructose. Disturbed hepatic GNG due to ALDOB failure may cause hypoglycemia in patients with HFI. If newborns and toddlers with HFI are not put on a fructose-free diet, they develop severe hepatic and renal injury, remain retarded in growth, and may die.

#### Fructose intolerance including fructose malabsorption due to malfunction of ChREBP

When ChREBP knockout mice or mice with isolated removal of ChREBP in the small intestine were kept on high-fructose diet, they showed a decreased small intestinal expression of Glut5 compared to wild-type mice and exhibited intestinal symptoms of fructose malabsorption such as dilatation of the intestine, diarrhea, and loss of weight [197, 230, 283]. The data suggest that ChREBP is critically involved in the d-fructose-dependent upregulation of GLUT5/Glut5. ChREBP is a transcription factor that is essential for adaption of metabolic programs in response to availability of carbohydrates, in particular to availability of d-fructose. ChREBP is upregulated by high-fructose diet [196]. It is critically involved in fructose-dependent stimulation of enzymes like ketohexose kinase (KHK), aldolase B (ALDOB), TRIOK, fructose-1,6-biphosphatase, glucose-6-phoshatase (G6PC), and lactate dehydrogenase (LDH) that are pivotal for fructolysis, gluconeogenesis, and/or lipogenesis (Fig. 8) [76, 170, 196, 197]. In the liver ChREBP, is supposed to be involved in emergence of dyslipidemia, metabolic syndrome, and NAFLD during high fructose consumption [3, 229, 241]. In genome-wide association studies, SNVs in ChREBP have been linked to hypertriglyceridemia, increased liver enzymes in the blood, and NAFLD [3]. Considering the role of ChREBP in d-fructose-dependent upregulation of GLUT5/Glut5 and of enzymes involved in fructolysis and GNG, it is expected that SNVs in ChREBP are associated with fructose malabsorption after fructose-rich meals.

## Effects of diabetes on glucose transporters in the small intestine

### General considerations

Considering the above-described effects of dietary d-glucose and of plasma insulin on expression and/or distribution of glucose transporters in the small intestine, it is expected that the expression and/or distribution of glucose transporters in the small intestine is changed during diabetes. This issue has been investigated in several animal models of diabetes and in humans.

### Effects of diabetes observed in animal models

#### Streptozotocin- or alloxan-induced type 1 diabetes mellitus in rodents

In the 1970s, it was reported that 4–140 days after treatment with alloxan or STZ that induce type 1 diabetes mellitus (T1DM) by destroying pancreatic β cells, the absorption of d-glucose or 3-ODG was increased [163, 246, 343]. Twenty to 140 days after alloxan or STZ treatment, small intestinal mass and villous surface area were increased [246, 343].

More than one decade later, the effects of STZ-induced T1DM on small intestinal glucose transporters were investigated in rats. In one study in which mRNA abundance of Sglt1 was determined 2–60 days after application of STZ, Sglt1 mRNA was increased after 30 days [264]. In another study, functional Sglt1 protein in rat small intestine was determined 14–60 days after application of STZ by measuring [^3^H]phlorizin binding [109]. In the jejunum, no effect on phlorizin binding was observed after 14 days; however, phlorizin binding was increased about 10-fold after 60 days. Additional studies on the effects of STZ on Sglt1-related immunoreactivity and on transport in rat small intestine were performed employing intact tissue and isolated BBMs [47, 83, 97, 218]. Whereas no upregulation of Sglt1 was detected 7 days after STZ application, upregulation of Sglt1-related immunoreactivity and Sglt1-mediated d-glucose transport was observed 2–6 weeks after STZ treatment.

Measuring mRNA of Glut2 and Glut5 in rat small intestine 2–60 days after STZ treatment, Glut2 mRNA abundance was increased after 2 days and reached a maximal level after 10 days whereas Glut5 mRNA was decreased after 10 days [264]. In contrast it was observed by another group that Glut5 mRNA in rat small intestinal mucosa was upregulated 7 days after STZ treatment [58]. Upregulation of Glut2 mRNA and Glut5 mRNA in rat small intestine was also observed 6 weeks after STZ application [47].

Corpe and coworkers investigated the effects of STZ on plasma membrane abundance and function of Glut2 and Glut5 in rat small intestine [70]. They measured transporter-related immunoreactivity in isolated BBMs and BLMs and determined d-fructose absorption employing small intestinal perfusion in vivo and in vitro. Five and 10 days after application of STZ, Glut2 was upregulated in the BLM and showed up in the BBM. In addition, Glut5 in the BBM was upregulated. The changes were associated with increased and decreased Glut5-mediated d-fructose absorption in vitro and in vivo, respectively. Whereas the increased absorption observed in vitro is consistent with the changed transporter concentrations in the BBMs and BLMs, the decreased d-fructose absorption observed in vivo may be due to changes of the intracellular carbohydrate metabolism. For example, it has been described that small intestinal expression of G-6-P was increased in STZ-induced diabetes [309]. Of note, STZ-induced upregulation of Sglt1, Glut2, Glut5, and G-6-P in the small intestine could be reversed by insulin [47, 218, 252].

#### Rodent models of type 2 diabetes

*Ob/ob* mice that do not express leptin, leptin receptor-deficient *db/db* mice, and Zucker diabetic fatty rats with a nonfunctional *db* receptor and a mutation affecting transcription in pancreatic β cells are frequently employed rodent models of type 2 diabetes mellitus (T2DM). In these diabetic models obesity, hyperglycemia, hyperlipidemia, hyperinsulinemia, and insulin resistance were observed [416].

Five to 10-week-old *ob/ob* mice are hyperphagic, obese, and hyperinsulinemic and exhibit hypertrophy and length increase of small intestine which persist in 20-week-old mice [90, 146, 272]. Absorption of 10 mM and 28 mM d-glucose per unit small intestinal length in 10 and 20-week-old ob/ob mice was higher compared to lean wild-type mice, whereas d-glucose absorption related to small intestinal weight was not different [272]. The data suggest that the involved transporters Sglt1 and Glut2 in the enterocytes are not upregulated. Consistently, a similar Sglt1-related immunoreactivity was observed in the BBM of 3–6-month-old *ob/ob* mice compared to heterozygous controls [90].

*Db/db* mice have a defective leptin receptor and increased plasma concentrations of leptin. Similar to *ob/ob* mice, *db/db* mice are hyperphagic, obese, hyperglycemic, and hyperinsulinemic [240, 416]. In 3–6-month-old *db/db* mice, small intestinal length and mucosal mass were increased due to cellular proliferation [90]. In small intestinal enterocytes of *db/db* mice at similar age, Sglt1 mRNA and total Sglt1 protein were increased [90, 240].

In 3–4-month-old obese Zucker diabetic fatty rats with large increased plasma glucose levels compared to lean wild-type mice, no differences in small intestinal mRNA and protein levels of Sglt1, Glut2, and Glut5 were detected [71].

### Effects of type 2 diabetes in humans

Expression and distribution of SGLT1, GLUT2, and GLUT5 in duodenal mucosal biopsies performed in the morning after overnight fasting were compared between patients with T2DM and healthy individuals at the same age [98]. The T2DM patients had a mean age of 58 years and most of them were on antidiabetic diets and underwent treatment with sulfonylurea drugs or metformin, whereas the control group received standard diet. In the duodenal mucosa of the T2DM patients, about 3-fold upregulation of SGLT1, GLUT2, and GLUT5 was observed on the mRNA level. In the BBM, only SGLT1- and GLUT5-related immunoreactivity was detected which was approximately 4-fold higher in the T2DM patients compared to the control group. The data suggest an upregulation of SGLT1, GLUT2, and GLUT5 and no BBM location of GLUT2 in treated T2DM patients between meals. The upregulation may be due to increased transcription.

In duodenal mucosal biopsies of newly diagnosed, so far untreated T2DM patients with a mean age of 71, a significant increase of L and K cells expressing SGLT1 was observed [393].

## Role of SGLT1 in enteric inflammation

### General considerations

#### Signaling in enterocytes during inflammation

The gastrointestinal tract contains a network of surveillance systems for host defense that include enterocytes, EECs, and immune cells with receptors recognizing pathogens [161, 291]. Signaling in IECs is mediated by nutrient components, commensal microflora, and pathogens that may induce intestinal inflammation. The pathogens include bacterial lipopolysaccharide (LPS), components of fungi, and protozoic parasites. Pathogenic signature structures are recognized by receptors including Toll-like receptors (TLRs) in IECs, EECs, and immune cells [8, 54, 291]. TLRs in IECs activate kinases including interleukin-1 receptor–associated kinases, MAP kinases, and PKC [235]. MAP kinases promote migration of NFκB into the nucleus where NFκB stimulates the expression of inflammatory cytokines [235, 275, 427]. Excreted cytokines activate and/or recruit immune cells like Th2 lymphocytes. Activated Th2 lymphocytes secrete interleukins that modulate immune response and downregulate the expression of TLRs in a feedback loop [273]. This may cause a permeability increase of the small intestinal epithelial cell layer. In addition, a decreased proliferation and increased apoptosis of IECs is observed during inflammation.

#### SGLT1-mediated signaling in enterocytes

Reflecting upon the role of SGLT1/Sglt1 in enteric inflammation, SGLT1/Sglt1-mediated signaling in the small intestine must be understood because cross-talk with inflammatory signaling is expected. Above, it has been described how membrane polarization by SGLT1/Sglt1 is supposed to induce trafficking of GLUT2/Glut2 to the BBM of IECs (Fig. 6). This includes activation of a voltage-dependent Ca^2+^ channel, activation of PKCβII, and phosphorylation of myosin II. It has been reported that SGLT1/Sglt1-mediated membrane depolarization also promotes recruitment of the Na^+^-H^+^-exchanger (NHE) 3 into the BBM; however, different intracellular signaling has been described. In this case, signaling involves phosphorylation of MAP kinase kinase 2 by p38 MAP kinase, downstream phosphorylation of RAC-beta serine/threonine-protein (Akt2) kinase, and phosphorylation of cytoskeletal linker protein ezrin by Akt2 [167, 292, 356]. Phosphorylated ezrin binds to both actin and the C-terminus of NHE3 which is supposed to promote the incorporation of NHE3 into the BBM [439].

### Effects of SGLT1 on enteric inflammation by bacterial lipopolysaccharide

#### Experiments with intestinal human cell lines

When monolayers of SGLT1-transfected Caco-2 cells were incubated with LPS in the presence of 5 mM d-glucose, DNA fragmentation, and caspase-3 cleavage indicating apoptosis and paracellular permeability were increased [432]. These effects were attenuated and the anti-apoptotic proteins B cell lymphoma (Bcl)-2 and Bcl-X(L) were increased when the treatment with LPS was performed in the presence of 25 mM d-glucose. The d-glucose-dependent cytoprotection was blunted in the presence phlorizin. Hence, it was concluded that an increased SGLT1-mediated glucose transport at high glucose concentrations represents a protective mechanism for LPS-induced apoptosis. Noteworthy, it was also observed that LPS promoted recruitment of SGLT1 to the plasma membrane at 25 mM d-glucose [433].

A protective SGLT1-dependent effect of glucose on LPS cytotoxicity was also observed in human colon carcinoma HT29 cells that express SGLT1 endogenously [292]. When HT29 cells that had been cultivated in the presence of 5 mM d-glucose were treated with LPS, a smaller release of IL-8/keratinocyte-derived chemokine and a blunted increase of nuclear migration of NFκB were observed compared to HT29 cells that had been cultivated with 1 mM d-glucose. The glucose effect on NFκB activation promoting apoptosis was dependent on phosphorylation of Akt2. The protective effects of d-glucose on apoptosis were abolished by phlorizin and reduced when the expression of SGLT1 was decreased by si-RNA technology. Noteworthy, the protective effect of d-glucose was independent from metabolism because it was also observed when the cultivation and LPS treatment was performed in the presence of 5 mM 3-OMG [292]. The data suggest that SGLT1-mediated glucose uptake affects LPS-induced intracellular signaling promoting apoptosis.

#### Experiments with mice

Protective effects of Sglt1-mediated d-glucose uptake into IECs on intestinal inflammation induced by oral application of LPS and on endotoxic shock by i.p. injection of LPS were demonstrated in mice [292]. After 5-day treatment with LPS by daily gavage, extensive damage of ICEs was observed that was due to apoptosis. The damage of the ICEs was prevented when a bolus of d-glucose or 3-OMG was applied together with LPS but not when the animals had been orally treated with phlorizin prior to gavage with LPS and d-glucose. To investigate the impact of Sglt1-mediated glucose uptake into ICEs on endotoxic shock, an animal model was employed in which a high dose of LPS was i.p. injected together with d-galactosamine [127]. When mice received 0.25 mg/kg LPS together with 1 g/kg d-galactosamine, a dramatic serum increase of tissue necrosis factor (TNF)-α and chemokine (C-X-C motif) ligand 1 (KC) was observed within 4 h and all animals died within 36 h [292]. However, when the animals were pretreated by gavaging with d-glucose or 3-OMG 1 h prior to the LPS/d-galactosamine injection, the increase of TNF-α and KC was largely reduced and the animals survived. The protective effects of d-glucose and 3-OMG were abolished when the animals had been pretreated with phlorizin. Noteworthy, no protective glucose effect on the LPS/d-galactosamine-induced endocytotic shock was observed after i.p. injection of d-glucose. The protection by glucose gavage was correlated with a 18-fold serum increase of the anti-inflammatory cytokine IL-10 4 h after the LPS/d-galactosamine treatment. The data indicate that the LPS/d-galactosamine-induced endotoxic shock initiated by small intestinal damage can be prevented by Sglt1-mediated d-glucose uptake independently of metabolism.

A dansyl C-glucoside (see called compound 5 in [220]) later on called BLF501 [53] was synthesized that is predicted to interact with Sglt1. BLF501 abolished the LPS-induced production of interleukin 8 (IL-8) in the human cell line HT29 similar to high d-glucose concentrations but was not effective when the expression of Sglt1 had been decreased by siRNA [220, 292]. These observations suggest that binding of BLF501 to SGLT1/Sglt1 triggers SGLT1/Sglt1-mediated intracellular signaling similar to SGLT1/Sglt1-mediated d-glucose cotransport. Noteworthy, the endocytotic shock after i.p. application of LPS and d-galactosamine was bunted when the animals had been gavaged with BLF501. Whereas all animals died within 24 h without pretreatment, all animals survived after BLF501 gavage [220].

### Effects of SGLT1 during infection with *giardia duodenalis*

*Giardia duodenalis* is a waterborne protozoan pathogen of humans and domestic animals that causes diarrhea worldwide [340]. Exposition with *giardia duodenalis* trophozoites induces structural and permeability changes in the small intestine due to increased apoptosis and caspase activity [293, 348, 392, 398]. Confluent cell layers of Caco-2 cells that had been transfected with rabbit Sglt1 were employed to investigate whether Sglt1-mediated d-glucose uptake has an impact on apoptosis after incubation with *giardia duodenalis* trophozoites or sonicates of the trophozoites [434]. After cultivation of the Caco2 cells with 5 mM d-glucose, a higher degree of caspase-3-dependent apoptosis was induced by *giardia duodenalis*–derived components compared to cultivation with 25 mM d-glucose. The protective effect of high d-glucose was dependent on Sglt1-mediated Na^+^-d-glucose cotransport during incubation with the *giardia duodenalis* sonicates since it was blunted when the incubation was performed in the presence of phlorizin. Of note, Sglt1-mediated AMG uptake into Caco2 cells and Sglt1 abundance in the plasma membrane were upregulated by *giardia duodenalis* sonicates. The data suggest that SGLT1/Sglt1-mediated membrane depolarization during d-glucose transport triggers signaling events that interact with the signaling cascade involved in *giardia duodenalis*–mediated apoptosis.

### Implication of SGLT1 on gastrointestinal mucositis during chemotherapy

Oral and gastrointestinal mucositis are serious side effects of many forms of radiotherapy and chemotherapy. Complex pathophysiologic processes are involved that include damaging of epithelial and immune cells and affect their complex interactions [364]. Changes in proliferation, apoptosis, and/or necrosis of IECs and changes in their cytoskeleton architecture were associated with drug-induced mucositis in the small intestine [53, 364]. Data obtained by two experimental setups suggest that SGLT1/Sglt1-mediated signaling influences effects of cytostatic drugs in the small intestine. Using confluent SGLT1 expressing LLC-PK_1_ cells grown in the presence of 5.6 mM d-glucose as model, it was observed that the electrical resistance of the cell layer was decreased and the percentage of necrotic cells was increased after 1-h incubation with cisplatin [172]. These effects were largely attenuated when the incubation with cisplatin was performed in the presence of phlorizin. Rumio and coworkers employed a mouse model in which intestinal mucositis was induced by i.p. injection of doxorubicin (DXR) or of DXR plus 5-fluorouracil (5-FU) and studied effects of oral application of the dansyl C-glucoside BLF501 on small intestinal morphology, on proliferation of IECs, and/or on a marker for apoptosis [53]. BL1501 is supposed to interact with SGLT1/Sglt1 and was shown to have a similar protective effect on LPS-induced endotoxic shock in mice as Sglt1-mediated glucose transport (see above). Seventy-two hours after a single i.p. injection of DXR, the number of proliferating IECs was reduced by 65% whereas the number of proliferating IECs was not altered when the animals were gavaged at the same time with BLF501 [53]. A three-times weekly i.p. application of DXR plus 5-FU resulted in dramatic morphological changes in the small intestine such as villus atrophy, reduction of villus length and loss of the BBM, and increase of caspase-3 expression. All these effects were not observed when the animals were gavaged with BLF501 during the DXR/5-FU injections. The experiments suggest that BLF501 activates SGLT1/Sglt1-mediated intracellular signaling which leads to protection of the IECs from DXR/5-FU injury.

## Small intestinal glucose transporters and therapeutic measures

### Oral rehydration therapy

Diarrheal diseases represent a global health threat in different societies. In developing countries, infection by bacteria, such as enterotoxigenic and enteropathogenic *E. coli*, *Salmonella*, *Shigella*, and *Vibrio cholerae*, and infection by various parasites cause millions of deaths. In industrial societies, infection by resistant bacteria and ingestion of food that is contaminated with bacterial toxins cause major problems. In 1968, it was discovered that oral ingestion of d-glucose-rich solutions could effectively replace intravenous infusion of large amounts of fluids to prevent life-threatening dehydration during cholera infection [157, 306]. After introducing and establishing the oral rehydration therapy (ORT) in developing countries for treatment of severe diarrhea caused by infections with *V. cholera*, rotavirus, *E. coli*, and *Yersinia*, countless lives were saved [312, 409]. The original oral rehydration solution contained 75 mM NaCl, 75 mM d-glucose, 20 mM KCl, and 10 mM sodium.

For diarrhea caused by various infections, effectiveness of ORT is mainly due to water absorption associated with SGLT1-mediated cotransport of sodium and d-glucose. Whereas SGLT1-mediated water uptake across the BBM has been studied in some detail, it is not understood how water reaches the intercellular space. In a first step, SGLT1-mediated Na^+^-d-glucose cotransport across the BBM promotes water uptake into the enterocytes. Expressing human SGLT1 in oocytes Loo and coworkers observed that SGLT1-mediated uptake of one molecule d-glucose was associated with uptake of 260 molecules of water [245]. They hypothesized that human SGLT1 translocates water together with Na^+^ and d-glucose. However, Lapointe and coworkers observed that SGLT1-mediated d-glucose uptake into oocytes expressing human SGLT1 precedes water uptake and showed that AMG uptake by SGLT1 generated a local osmotic gradient that is sufficient to drive passive water influx across the BBM [96, 126, 226]. Measuring the water permeability of confluent monolayers of MDCK cells transfected with human SGLT1, it was observed that passive water permeability was orders of magnitude larger than water permeability associated with SGLT1-mediated glucose uptake [105]. Under the restriction that MDCK cell monolayers may not represent IEC layers sufficiently well, this observation suggests that transcellular water movement is small and cannot explain the effect of ORT. Most probably, SGLT1-mediated increase of sodium and d-glucose in IECs stimulates efflux of sodium and d-glucose across the lateral membranes via the Na, K-ATPase, and GLUT2, respectively. The high osmolarity in the intracellular spaces is supposed to promote water flux across the tight junctions [228, 367].

The oral rehydration solution proposed by the WHO has been modified by recommending a reduced osmolarity and a smaller concentration of d-glucose [360]. In addition, it has been proposed to include starch and Zn^2+^ [33, 164, 359]. Discussing the role of SGLT1 for oral rehydration during diarrhea caused by infections, it should be considered that sodium uptake into enterocytes is also mediated by the Na^+^-H^+^-exchanger NHE3 and that infections by enteropathogenic *E. coli* and rotavirus are associated with a decreased expression of SGLT1 in enterocytes [80, 82, 144].

### Parenteral nutrition

Parenteral nutrition is a life-saving medical measure that is employed in premature babies with incompletely developed intestinal functions and in adults during medical treatments of prolonged coma, sepsis, pancreatitis, intestinal malabsorption, short bowel syndrome, and severe burns. Considering the various regulations of small intestinal functions in response to luminal nutrients, effects of parenteral nutrition on small intestinal glucose absorption are expected. Consistently, it was observed in rats, piglets, and/or humans that intestinal mass, intestinal surface area, and villi length were decreased during parenteral nutrition [41, 48, 73, 138, 181, 234]. Evidence was provided that also small intestinal absorption of amino acids and d-glucose were decreased [73, 174, 207]. The effects on transport are mainly due to the changes in intestinal morphology and intestinal mass. For example, after parenteral nutrition, absorption of 3-OMG per centimeter of the small intestine was smaller compared to enteral nutrition whereas 3-OMG absorption per gram of the small intestine was larger [207]. After parenteral nutrition, the blood concentration of enterohormones such as GLP-2, GIP, and PYY were lower compared to enteral nutrition [48]. Consistent with established trophic effects of GLP-2 on the small intestine [94], evidence has been presented that application of GLP-2 or a GLP-2 agonist during parenteral nutrition reduces atrophy in the small intestine [73, 180, 346].

### Treatment of type 2 diabetes with metformin

Oral application of metformin influences d-glucose metabolism in the small intestine and changes d-glucose absorption and basolateral d-glucose uptake into intestinal tissue. After oral application of metformin to rats, an increased small intestinal glucose utilization was observed [22, 304]. In mice, absorption of 2-deoxy-2-[^18^F]-d-glucose (2-[^18^F]DG) was decreased after a single oral application of metformin [165]. Consistently, positon emission tomography (PET) in mice gavaged with 2-[^18^F]DG revealed that the absorption of 2-[^18^F]DG was decreased after oral application of metformin [165] (L. Zubiaga and F. Francois, unpublished data). After short- and/or long-term oral application of metformin to rats and mice, uptake of i.v. applicated 2-deoxy-d-[^3^H]-glucose or 2-[^18^F]DG into small intestinal tissue was increased [22, 165, 201]. In metformin-treated T2DM patients undergoing PET with i.v. injected 2-[^18^F]DG, a higher intensity of PET signal in the small intestine was observed compared to T2DM patients that were not under metformin treatment [201, 287].

Some data from rodents concerning effects of metformin on abundance of Sglt1 and Glut2 in the small intestinal BBM have been reported. In the absence of luminal d-glucose, metformin decreased the BBM abundance of Sglt1 in rat small intestine whereas it increased the BBM abundance of Glut2 [335]. After luminal application of 10 mM d-glucose in the absence of metformin, the abundance of both Sglt1 and Glut2 in the BBM was increased. Metformin decreased the elevated BBM abundance of Sglt1 in the presence of d-glucose, whereas it further increased the elevated BBM abundance of Glut2 in the presence of d-glucose. Consistently, Sglt1-mediated short-circuit current induced by 10 mM luminal d-glucose was decreased after a 3-min preincubation with metformin [335]. The data indicate a differential interference of metformin with glucose-dependent intracellular trafficking of Sglt1 and Glut2 to the BBM that may lead to a decrease or increase of d-glucose uptake at different glucose concentrations.

Whereas the metformin-induced upregulation of basolateral uptake of 2-DOG and 2-deoxy-2-F-d-glucose (2-FDG) into IECs may be due to an increase of Glut2 abundance in BLM, it is not understood why metformin decreases the absorption of 2-DOG and 2-FDG. This effect is probably independent of Sglt1 and Glut5 because these transporters do not accept 2-DOG and 2-FDG as substrates. It suggests downregulation of an additional transporter in the BBM that is critical for absorption of DOG and 2-FDG.

Recently, it was observed in mice that oral application of metformin blunted the increase of plasma d-glucose during OGTTs and that this effect was abolished in Sglt1 knockout mice but persisted in Glut2 knockout mice [165] (L. Zubiaga and F. Francois, unpublished data). These data may be explained by the requirement of Sglt1-mediated depolarization of the BBM for upregulation of Glut2 and/or a unidentified glucose transporter in the BBM and/or BLM.

### Bariatric surgery

#### Surgery procedures

**Fig. 9:**
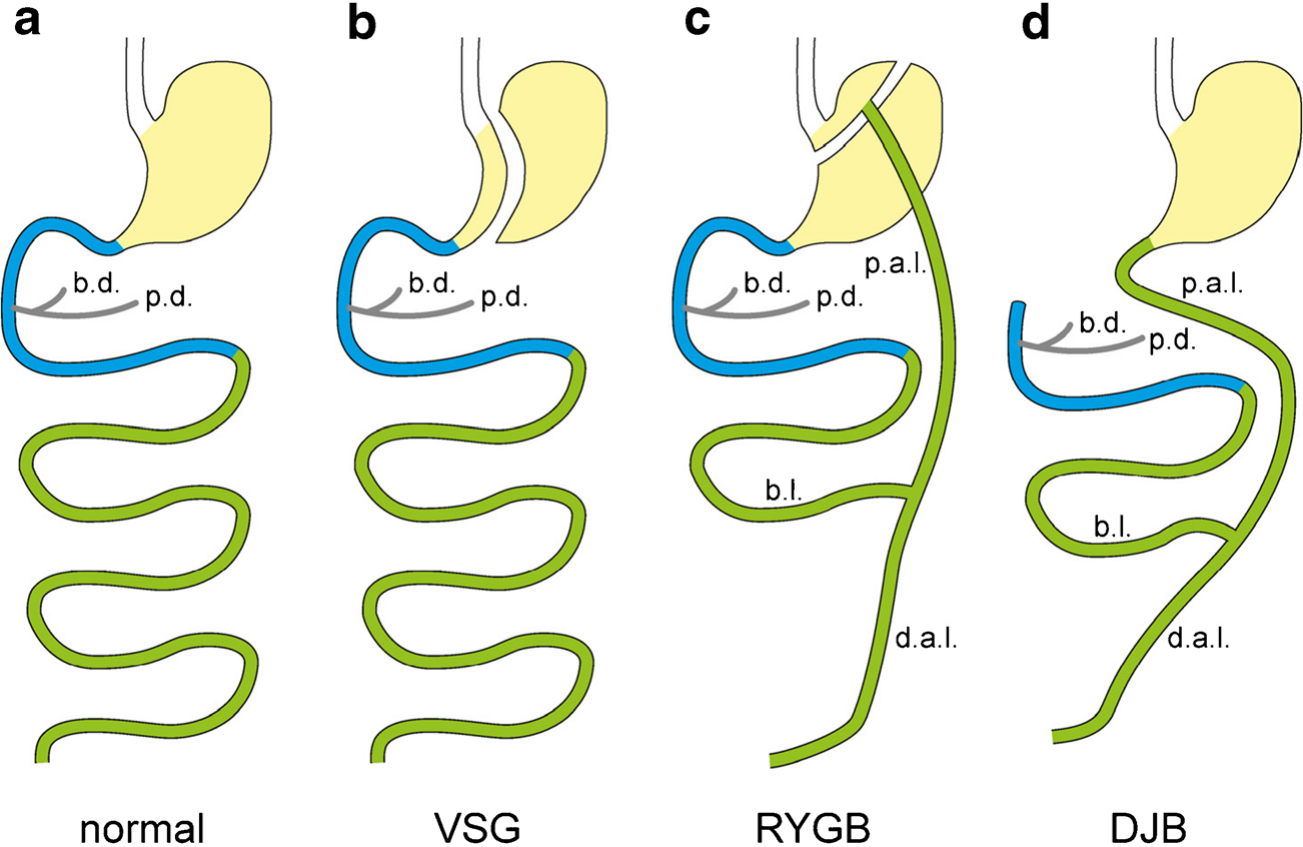
Schematic representation of the most common bariatric surgery procedures. **a** Normal situation. **b** VSG, vertical sleeve gastrectomy. **c** RYGB, Roux-en-Y gastric bypass. **d** DJB, duodeno-jejunal bypass. Stomach yellow, duodenum blue, jejunum green. b.d, bile duct; p.d., pancreatic duct; p.a.l., proximal alimentary limb; d.a.l., distal alimentary limb; b.l., bile limb

Bariatric surgery procedures have been approved as most effective medical measures to reduce body weight of morbidly obese individuals improving follow-up diseases such as heart attack, stroke, and cancer [42, 310, 342]. Bariatric surgery procedures turned out to improve T2DM independently of body weight reduction [263, 298, 329, 342]. Different surgery procedures are employed; however, vertical sleeve gastrectomy (VSG) and Roux-en-Y gastric bypass (RYGB) are predominantly applied (Fig. 9) [342, 366]. In VSG, most of the stomach is removed leaving a vertical tube. Hence, ingested food is not acidified and partially degraded in the stomach and rapidly delivered to duodenum. In addition, gastric regulation of bile secretion, pancreatic secretion, and appetite are abolished. In RYGB, the stomach is dissected in the very upper part. The lower part of the stomach is closed whereas the upper part is connected to the lower end of the jejunum that is dissected in its top third. The upper part of the dissected jejunum that contains the junction of the pancreatic/bile duct, is connected end to side to the distal end of the jejunum. Hence, proximal and distal alimentary limbs and a bile limb of the jejunum can be distinguished (see p.a.l., d.a.l., b.l. in Fig. 9c). Investigating the underlying mechanisms of bariatric surgery in animal models, two additional surgery procedures, duodeno-jejunal bypass (DJB) (Fig. 9d) and ileal interposition (IIP), have been studied. DJB can be considered modification of RYGB in which the removal of the stomach is omitted. It is probably less effective concerning body weight reduction and antidiabetic effect compared to RYGB [438]. In IIP, a segment of the distal ileum is interposed into the proximal jejunum exposing jejunal L cells to high nutrient concentrations [20, 184, 297].

#### Proposed functional mechanisms

Various functional mechanisms are supposed to contribute to weight reduction and/or weight-independent antidiabetic effects of bariatric surgical procedures [160, 221, 349]. The complete panel of proposed effective mechanisms comprising five main groups are expected in RYGB. First, appetite regulation and/or energy expenditure may be changed due to omission of gastric neuronal and ghrelin-mediated signals to the brain in response to stomach removal (VSG) or to separation of the stomach from alimentary passage (RYGB). Second, small intestinal absorption of nutrients including monosaccharides that may influence energy balance, carbohydrate metabolism, and/or composition of the microbiome may be decreased or slowed down. This may be due to shortening of the alimentary path, to morphological changes in the small intestine, to decreased expression and functional activity of small intestinal nutrient transporters and/or digestive enzymes, and to the absence of bile acids and pancreatic enzymes in parts of the alimentary path. Third, the stimulation of enterohormone secretion by L cells (GLP-1, GLP-2, and PYY) and K cells (GIP) may be changed [206, 289, 298, 310, 415]. Whereas K cells are mainly located in the jejunum, L cells are mainly located in the ileum and colon [317, 320]. Because SGLT1/Sglt1 expressed in L and K cells is critically involved in d-glucose-dependent stimulation of enterohormone secretion by these cells, the concentration of d-glucose in the jejunum and ileum has a critical impact on enterohormone secretion. In particular, evidence has been provided that the secretion of GLP-1 during OGTTs is increased when the d-glucose concentration in the ileum is increased by reducing absorption of d-glucose in the jejunum during RYGB or DJB [183] or by an increased d-glucose concentration in the ileum by IIP [184]. Fourth, the concentrations of bile acids in part of the alimentary tract is decreased changing lipid absorption and metabolism [118, 299, 333]. Fifth, the composition of the gut microbiome is changed with impact on metabolic functions in the small intestine and the entire organism [349].

#### Effects on intestinal structure and glucose transporters

Alimentary-mediated stimulators trigger enterohormone-mediated and/or nervous signals that promote proliferation and differentiation of small intestinal mucosa [352]. Hence, small intestinal segments that are excluded from contact with nutrients such as the bile limbs in RYGB and DJB or from contact with enzymatically digested nutrients such as the proximal alimentary limbs in RYGB and DLB undergo morphological changes [209, 426]. These changes may be atrophic or hyperplastic including a decrease or increase of the mucosal surface, respectively. In addition, the expression and plasma membrane location of glucose transporters may be changed.

After RYGB and/or DJB in healthy and diabetic rodents, mucosal surface area and villi length in the bypassed foregut were decreased which was combined with a reduction of Sglt1-mediated AMG uptake per intestinal length [183, 209, 290, 426]. In the proximal alimentary limbs after RYGB and GJB receiving bile-less undigested food, mucosal hypertrophy with increased mucosal surface area, villi length, and increased number of L cells was observed [44, 59, 145, 274, 334, 368, 429]. In rodents, also the glycolytic metabolism was increased [334]. Noteworthy, these changes were associated with downregulation of Sglt1 in the BBM and with upregulation of Glut1 in the BLM. The abundance Sglt1 mRNA and of Sglt1-mediated AMG uptake per intestinal length were decreased [183, 429]. After RYGB in rats, uptake of i.v. applied 2-[^18^F]DG into the proximal alimentary limb was increased and the expression of Glut1 was increased whereas the expression of Glut2 was not changed [59, 334].

One month after VSG in mice, the body weight of the animals was reduced and the increase of blood glucose after OGTT was blunted [426]. In the jejunum, Sglt1 mRNA and abundance of Sglt1 protein in the BBM were decreased [426].

The duodenum and jejunum have a larger mucosal surface with longer villi compared to the ileum. When in IIP in normal and diabetic rats, the distal ileum was transposed into the proximal jejunum, hypertrophy of the transposed segment was observed [184, 202, 204]. Thereby, ileal mass including protein and DNA, mucosal surface, and villi length were increased. Sglt1 protein in the BBM and phlorizin-inhibited AMG uptake per unit length were increased so that the capacity of Sglt1-mediated glucose uptake was similar to the surrounding jejunum [184].

The data suggest that differential impacts of bariatric surgery procedures on expression of SGLT1/Sglt1 and/or GLUT1/Glut1 contribute to antidiabetic and/or antiobese effects. However, since bariatric surgery acts via various mechanisms that appear to be effective in different combinations, effects of bariatric surgery procedures on small intestinal glucose transporters may be supportive rather than critical for the achieved therapeutic effects.

## Glucose transporters in the small intestine as drug targets

### Food compounds

#### Special foods and food extracts

Antidiabetic properties have been associated with foods containing flavonoids and other phenolic compounds such as strawberries and blueberries, yerba maté, and germinated waxy black rice [68, 141, 186, 285, 328, 419]. Evidence has been presented that such foods or extracts thereof inhibit and/or downregulate small intestinal glucose transporters. Thus, anthocyanin-rich berry extracts acutely inhibited SGLT1- and GLUT2-mediated glucose transport [11, 107, 256]. In addition, berry extracts downregulated SGLT1 mRNA, GLUT2 mRNA, and GLUT2 protein within 12–16 h [11]. When normal rats or rats with alloxan-induced T1DM were gavaged during 4 weeks with extract of Yerba maté (*Ilex paraguariensis*), the expression of Sglt1 mRNA in the small intestine related to mRNA of β-actin was decreased [285]. Furthermore, the expression of Sglt1 and Glut2 in rats with STZ-induced T1DM was decreased on mRNA and protein levels when the food was supplemented for 8 weeks with germinated waxy black rice [186]. Although specific foods or food extracts may have beneficial effects, usage of the effective food components is preferred for practical and security reasons.

#### Isolated food compounds

Interactions of individual flavonoids and the glucose analogue 1-deoxynojirimycin (DNJ) present in mulberry leaves with SGLT1/Sglt1, GLUT2/Glut2, and/or GLUT5/Glut2 were tested. The measurements were performed using *Xenopus laevis* oocytes in which the respective transporters were expressed and in preparations of rat intestinal mucosa.

The flavonol quercetin is present in various foods such as red wine, onions, and green tee [328]. In foods, quercetin mostly occurs in glycosylated forms; however, it is deglycosylated in the small intestine [219]. In the small intestine, quercetin concentrations of up to 100 μM were determined; however, since absorption of quercetin is poor, the plasma concentration does not exceed 2 μM [219]. Measuring substrate-induced inward currents in voltage-clamped oocytes expressing human SGLT1, no SGLT1-mediated transport of unglycosylated or glycosylated quercetin was detected [208]. When quercetin was glycosylated at C3 or C4, SGLT1-mediated uptake of AMG was inhibited with apparent *K*_i_ values of 640 μM and 40 μM, respectively, whereas incomplete low affinity inhibition of human SGLT1 was observed with nonglycosylated quercetin [208]. Hence, inhibition of SGLT1 in the small intestine by quercetin does not appear to be of biomedical relevance. However, uptake of 2-DOG and d-fructose by human GLUT2 expressed in oocytes was inhibited by quercetin with *IC*_50_ values of 13 μM and 16 μM, respectively [219]. A similar inhibition was observed with the quercetin analog myricetin whereas less affine or no inhibition was observed with glycosylated forms of quercetin. Uptake of d-glucose or d-fructose in oocytes expressing human GLUT5 were inhibited neither by nonglycosylated quercetin nor by glycosylated quercetin and myricetin. Testing the acute effect of quercetin on an OGTT in diabetic Zucker *fa*/*fa* rats, it was observed that quercetin blunted the increase of plasma d-glucose during the OGTT [363]. The data suggest an antidiabetic effect of quercetin after ingestion of high amounts of d-glucose or d-fructose when GLUT2/Glut2 is targeted to the BBM.

In Ussing chamber experiments with mouse jejunum, glucose-dependent transmucosal currents mediated by Sglt1 were decreased within few minutes after mucosal application of the anthocyanin flavonoid delphinidin [154]. Delphinidin also decreased 3-OMG absorption in mouse small intestine and in Caco2 cell that was correlated with oscillations of intracellular Ca^2+^ concentrations. In addition, data were reported suggesting that the delphinidin-mediated decrease of 3-OMG absorption in Caco-2 cells involves activation of fatty acid receptor GPR40. It was not resolved whether delphinidin inhibited SGLT1/Sglt1 or downregulated the abundance of SGLT1/Sglt1 in the BBM and whether effects on GLUT2/Glut2 were involved.

1-Deoxynojirimycin (DNJ) is a glucose analogue occurring in mulberry leaves that inhibits α-glucosidases and modulates hepatic metabolism [216, 237]. Effects of DNJ on OGTTs and on the amounts of Sglt1 in the BBM and of Glut2 in the BLM of IECs were investigated in healthy mice and in mice with STZ-induced T1DM [239]. DNJ was orally applicated for 3 days, twice a day, and 15 min before the OGTTs were started. In DNJ-treated mice, the increase of blood glucose during the OGTTs was blunted and the abundance of Sglt1 in the BBM and of Glut2 in the BLM after the OGTTs was decreased. It was not resolved whether the effects of DNJ on membrane abundance of Sglt1 and Glut2 were secondary to inhibition of Sglt1 or whether DNJ enters the enterocytes and exhibits intracellular effects on transporter regulation.

### Synthetic compounds

#### SGLT1 inhibitors

In addition to selective SGLT2 inhibitors, dual inhibitors of SGLT1 and SGLT2 and selective SGLT1 inhibitors have been developed for oral treatment of T2DM. Whereas SGLT2/Sglt2 inhibitors reduce the reabsorption of ultrafiltrated d-glucose in the kidney, inhibitors of SGLT1/Sglt1 decrease small intestinal d-glucose absorption. Sotagliflozin (LX4221) [227, 277, 436, 437] and licogliflozin [150] are effective dual inhibitors whereas mitagliflozin [175] and compounds GSK-1614235 [88], LX2761 [131], and SGL5213 [176, 217] inhibit SGLT1/Sglt1 in the small intestine selectively. Mitagliflozin and GSK-1614235 are absorbed; however, they inhibit SGLT1/Sglt1 with a much higher efficacy compared to SGLT2/Sglt2. LX2761 and SLC5213 inhibit SGLT1/Sglt1 and SGLT2/Sglt2 with similar efficacies but enter the blood only slowly so that their systemic concentrations are not effective [131, 217]. Selective inhibition of SGLT1/Sglt1 in the small intestine may induce three main effects. First, by inhibiting SGLT1/Sglt1 in IECs d-glucose absorption may be reduced. Second, a decreased absorption in the duodenum and proximal jejunum may lead to an increase of the d-glucose concentration in the distal jejunum and in the ileum where most L cells are located and may thereby increase d-glucose-dependent secretion of GLP-1 in L cells and promote pancreatic insulin secretion. Third, since glucose-dependent GLP-1 secretion in L cells is mediated by SGLT1/Sglt1, inhibition of SGLT1/Sglt1 in L cells may blunt GLP-1 secretion. Antidiabetic effects of selective SGLT1/Sglt1 inhibitors have been demonstrated in rodents. In normal rats, in rats with STZ-induced T1DM, and in normal mice, it was observed that GSK-1614235, LX2761, and/or SGL5213 decreased postprandial elevation of blood glucose [88, 131, 176]. However, no consistent effects of the SGLT1/Sglt1 inhibitors on postprandial increase of GLP-1 in the blood were observed.

#### Compounds that downregulate SGLT1

A downregulation of SGLT1 abundance in the small intestine is considered an attractive alternative to inhibition of SGLT1 because longer lasting effects may be achieved. In addition, a selective downregulation of SGLT1 in IECs versus L cells and/or downregulation under defined physiologic or pathophysiologic conditions may be possible. Oral application of specific SGK1 inhibitors may be used to downregulate the increased expression SGLT1 in diabetic patients whereas modified peptides derived from the regulatory domain of RS1 may be employed to prevent the rapid upregulation of SGLT1 in the small intestinal BBM after ingestion of glucose-rich food.

SGK1 is ubiquitously expressed and under genomic control of hormones including growth factors, corticosteroids, and insulin [224]. It participates in the regulation of various transporters, ion channels, and enzymes and is involved in pathophysiological changes during obesity, diabetes, hypertension, and tumor growth [223]. Under normal physiological conditions, the expression of SGK1 is low; however, it is increased under certain pathophysiological conditions such as hyperglycemia and ischemia. In obese and diabetic individuals, an increased SGK1 expression was observed in several tissues including small intestine [238]. In the small intestine of diabetic *db/db* mice, higher mRNA and protein abundance of SGK1 and Sglt1 was observed compared to nondiabetic control mice [240]. After oral application of the selective SGK1 inhibitor EMD638683 for 8 weeks to *db/db* mice, Sglt1 mRNA and Sglt1 protein in the small intestine were decreased and fasting blood glucose was reduced. Experiments performed with cultivated rat intestinal IEC-6 cells revealed that dexamethasone treatment increased the expression of SGK1 and Sglt1 and that the expression and function of Sglt1 could be downregulated by incubation with EMD638683. The data suggest that oral application of selective SGK1 inhibitors that do not enter the systemic circulation may be employed for antidiabetic therapy (see effect of SGK1 on transcription of SGLT1 in Fig. 2).

Modified peptides derived from the regulatory domain of RS1 (RS1-Reg) may be used to prevent the glucose-dependent, short-term upregulation of SGLT1 in the small intestinal BBM [200]. The proposed mechanism is depicted in Fig. 4. In IECs, the release of SGLT1 containing vesicles from the Golgi is promoted by the enzymatic activity of ODC. At low intracellular d-glucose concentrations, the vesicle release is slowed down because RS1-Reg binds to ODC and blocks the enzymatic activity. After uptake of glucose-rich food when the d-glucose concentration in IECs increases, d-glucose binds to ODC. Thereby, a conformational change is induced that decreases the efficacy of RS1-Reg binding and prevents the inhibition of ODC. This promotes the release of SGLT1-containing vesicles from the Golgi and leads to the increase of SGLT1 in the BBM. Of note, it was observed that injection of a RS1-Reg variant in which serine in a QSP motif was replaced by glutamate (RS1-Reg(QEP)) into oocytes expressing human SGLT1 promoted highly efficient downregulation of SGLT1-mediated AMG uptake in the presence of high intracellular glucose concentrations [341, 408] (see Fig. 4(C)). After oral application of RS1-Reg(QEP) to mice and subsequent oral gavage with a high amount of d-glucose, a decrease of glucose absorption and of Sglt1 abundance in the small intestinal BBM was observed [408]. In these experiments, RS1-Reg(QEP) had been linked to nanohydrogels to promote uptake into IECs. Of note, it was also observed that QEP promoted downregulation of SGLT1-mediated uptake in oocytes expressing human SGLT1 at high intracellular glucose concentrations in contrast to QSP [341]. After oral application of QEP for 3–6 days to diabetic *db*/*db* mice, antidiabetic effects were observed (, C. Otto, A. Friedrich, I. Vrhovac Madunić, C. Baumeier, R. W. Schwenk,  A. Karaica, C.-T. Germer, A. Schürmann, I,. Sabolić, H. Koepsell, unpublished data). Thus, fasting plasma glucose was decreased, insulin sensitivity was increased, the increase plasma glucose in OGTT was blunted, and the secretion of GLP-1 in the OGTT was increased. So far it has not been elucidated whether QEP also downregulates SGLT1/Sglt1 in L cells. Future studies are necessary to improve drug formulation in order to accelerate uptake into IECs.

## Conclusions

Extensive research has been performed concerning involvement of transporters and their regulation in monosaccharide absorption and impact of monosaccharide transporters on diseases and drug treatment. The data indicate a great complexity of these biomedically important issues and show that they demand further comprehensive in-depth investigation. For example, besides GLUT2, GLUT5, and SGLT1, several additional glucose transporters are expressed in the small intestine, the roles of which have not been investigated in appropriate detail. Regarding previous studies on GLUT2, GLUT5, and SGLT1, often short- and long-term post-translational regulations have not been distinguished unambiguously. Also, the functional mechanisms of most described regulatory processes of glucose transporters in ICEs have not been resolved in detail. In addition, most observations were made in rodents and have not been verified in humans. In view of overeating with carbohydrate-rich food and associated obesity with follow-up diseases in industrial societies, it is a challenge to develop drugs that downregulate small intestinal glucose transporters selectively in IECs. More detailed understanding on how all human glucose transporters expressed in small intestine function and are regulated in health and disease should be the basis for future dietary commendations.

## Abbreviations

Akt2: RAC-beta serine/threonine-protein
ALDOB: Aldolase B
AMG: α-Methyl-d-glucoside
AMPK: AMP-stimulated protein kinase
BBB: Brush border membrane
BLM: Basolateral membrane
BMAL: Brain and muscle arnt-like protein
CBP: cAMP response element protein–binding protein
CCK: Cholecystokinin
ChREBP: Carbohydrate-responsive element–binding protein
CRE: cAMP response element
CREB: cAMP response element–binding protein
DJB: Duodeno-jejunal bypass
DNJ: 1-Deoxynojirimycin
2-DOG: 2-Deoxy-d-glucose
DXR: Doxorubicin
EEC: Enteroendocine cell
EGF: Epithelial growth factor
EGFR: Epithelial growth factor receptor
ER: Endoplasmic reticulum
FBS: Fanconi-Bickel syndrome
2-[^18^F]DG: 2-Deoxy-2-[^18^F]-d-glucose
FGID: Functional gastrointestinal disorder
5-FU: 5-Fluorouracil
GIP: Glucose-dependent insulinotropic hormone
GLP-1: Glucagon-like peptide 1
GLP-2: Glucagon-like peptide 2
GGM: Glucose-galactose malabsorption
GNG: Gluconeogenesis
HFI: Hereditary fructose intolerance
HNF: Hepatic nuclear factor
HSP: Heat shock protein
HuR: Human antigen R
IEC: Intestinal epithelial cell
IFM: Isolated fructose malabsorption
IIP: Ileal interposition
KHK: Ketohexokinase
LXR: Liver X receptor
LPS: Lipopolysaccharide
MAP: Mitogen-activated protein
NAFLD: Nonalcoholic fatty liver disease
NHE: Na+–H+ exchanger
ODC: Ornithine decarboxylase
OGTT: Oral glucose tolerance test
ORT: Oral rehydration therapy
3-OMG: 3-O-Methyl d-glucoside
PET: Positon emission tomography
PPAR: Peroxisome proliferator–activated receptor
PYY: Peptide YY
RELM-β: Resistin-like molecule beta
RYGB: Roux-en-Y gastric bypass
SNV: Single nucleotide variation
STZ: Streptozotocin
TGFβ1: Tissue growth factor beta 1
SGK: Serum- and glucocorticoid-inducible kinase
T1DM: Type 1 diabetes mellitus
T2DM: Type 2 diabetes mellitus
TGN: Trans-Golgi network
TLR: Toll-like receptor
TRIOK: Triosekinase
URE: Uridine-rich element
YFP: Yellow fluorescent protein
VSG: Vertical sleeve gastrectomy
